# scDecorr: feature decorrelation based representation learning enables self-supervised alignment of multiple single-cell experiments

**DOI:** 10.1038/s41598-026-50586-z

**Published:** 2026-04-29

**Authors:** Ritabrata Sanyal, Yang Xu, Hyojin Kim, Rafael Kramann, Sikander Hayat

**Affiliations:** 1 Department of Medicine 2, RWTH Aachen University, Medical Faculty, Aachen, Germany; 2 Data Sciences Platform, Broad Institute of MIT and Harvard, Cambridge, MA USA; 3 Cardiovascular Research Institute, Department of Medicine, Icahn School of Medicine at Mount Sinai, New York, USA; 4 Windreich Department of Artificial Intelligence and Human Health, Icahn School of Medicine at Mount Sinai, New York, USA

**Keywords:** Single-cell transcriptomics, Self-supervised learning, Feature decorrelation, Data integration, Computational biology and bioinformatics, Mathematics and computing

## Abstract

Single-cell RNA sequencing (scRNA-seq) has revolutionized our understanding of cellular heterogeneity in complex biological systems. However, analyzing and integrating scRNA-seq data poses unique computational challenges due to sparsity, high variability, and technical batch effects. Here, we propose a novel framework called scDecorr for robust representation learning and data integration for scRNA-seq analysis. Our approach leverages the idea of feature decorrelation-based self-supervised learning (SSL) to obtain efficient low-dimensional representations of individual cells without relying on cell-type annotations. By maximizing similarity among distorted embeddings while decorrelating their components, scDecorr captures the biological signature while eliminating technical noise. Furthermore, scDecorr incorporates unsupervised domain adaptation to bridge the gap between batches with different distributions, enabling effective integration of scRNA-seq data from diverse sources. Our framework achieves domain-invariant representations by learning cell embeddings independently across domains and employing domain-specific batch normalization. We evaluate scDecorr on a variety of single-cell datasets and demonstrate its ability to integrate batches without losing the inherent biological variance, thereby facilitating optimal clustering. The representations generated by scDecorr also exhibit robustness in label transfer tasks, allowing for effective transfer of cell-type labels from reference to query datasets. Overall, scDecorr offers a powerful tool for efficient analysis and integration of large and complex scRNA-seq datasets, advancing our understanding of cellular processes and disease mechanisms. The code is available here https://github.com/hayatlab/scdecorr.

## Introduction

Single-cell RNA sequencing (scRNA-seq) has emerged as a powerful tool to study cellular heterogeneity in complex biological systems^1–3^ by enabling the measurement of gene expression at the individual cell level. However, scRNA-seq data presents unique computational challenges, including sparsity and high variability, which can adversely affect the accuracy of cell clustering and detection of rare cell populations. In this regard, obtaining a reliable low-dimensional representation for each cell that preserves the biological signature while eliminating technical noise is a crucial step in scRNA-seq data analysis^4,5^. Furthermore, integrating scRNA-seq data from different sources, batches and platforms poses a significant challenge due to technical batch effects, which can confound the inherent biological variations^6–8^. These confounders can also influence downstream data analysis and interpretation. Adding to these challenges, cell-type labels, which provide an important learning signal, are often unavailable or noisy. Various computational tools and pipelines, including Seurat^9^, Harmony^10^, MNN-Correct^11^, scVI^12^ have been developed to address these challenges. With the increasing availability of large and complex scRNA-seq datasets, there is a pressing need for accurate and efficient methods to analyze and integrate these data to improve our understanding of cellular processes and disease mechanisms.

The task of identifying an efficient low-dimensional, cell class discriminative, batch-invariant representations for single cells can be concisely defined as follows: the representation learning algorithm is required to map the gene expression profile onto a low-dimensional embedding, such that functionally similar cells are in close proximity while maintaining a distance between dissimilar cells. Furthermore, the model must be resistant to batch effects, so that gene expression profiles belonging to the same cell type, regardless of their batch origin, are assigned identical locations in the low-dimensional space.

Recently, self-supervised learning (SSL) has gained significant traction for learning robust representations across various domains–including computer vision and natural language processing (NLP)–by solving carefully designed pretext tasks^13–17^. Unlike supervised approaches, SSL methods do not rely on labeled data; instead, they learn from the data itself through pretext tasks such as masked image modeling, rotation prediction, or contrastive instance discrimination. In particular, contrastive learning (CL), a branch of SSL has emerged as a promising approach for finding robust low-dimensional single-cell representations^18–22^. CL enables representation learning by distinguishing positive pairs from negative pairs, where positive pairs consist of semantically similar transcriptional profiles, and negative pairs consist of dissimilar ones. CL-based techniques construct positive cell pairs and negative cell pairs with the aim of concentrating positive pairs and separating negative pairs using a contrastive loss^23^. There are primarily two main strategies for constructing these pairs. The first strategy^20,21,24^ involves computing inter-batch mutual nearest neighbors and intra-batch nearest neighbors for constructing positive pairs. The negative pairs are usually constructed by random sampling. This strategy introduces an overhead of computing the nearest neighbors in every iteration of the training run. The second strategy generates positive pairs of an anchor cell gene profile via random augmentations and constructs negative pairs by random sampling from a memory bank^25,26^ or by using the current mini-batch^13,22^. These methods typically require a large number of negative samples to generate high-quality representations and thus have a large memory footprint. Moreover, these techniques often also suffer from the problem of false negatives^27^. In the absence of labeled data, there is a chance that the anchor cell may form a negative pair with a sample from the same class, which reduces the contribution to the contrastive loss, limiting the model’s ability to converge quickly. Moreover, in situations where samples originate from various sources or batches, the contrastive loss may erroneously treat all samples from different sources as negatives, despite them belonging to the same cell class. This failure to differentiate between domains can widen the gap between batches^27^, which could result in the inability to learn batch-invariant representations, thus hindering data integration performance.

Despite recent advances, the use of negative samples continues to pose fundamental challenges to CL. As a result, there has been an increasing interest in self-supervised learning methods that do not rely on negative samples, paving the way for further research in this area^16,28–30^. Recently, feature decorrelation methods^30–32^ were proposed in SSL to facilitate learning representations without using negative samples. The objective of these methods is to learn a decorrelated embedding space by maximizing the information content of the embeddings^30^. This prevents information collapse in the embedding variables which contain redundant information. Inspired by the efficacy of feature decorrelation based SSL in modelling unlabelled data without negative pairs^30^, we expect that effective low-dimensional single cell representations can be obtained by decorrelating the different vector components of cell embeddings. However, it should be noted that using feature decorrelation-based SSL frameworks directly to discover robust representations for single cell gene profiles is sub-optimal when dealing with data from multiple sources or batches, as it fails to align them effectively. To achieve better data integration performance, we use feature decorrelation SSL coupled with unsupervised domain adaptation^27,33^. The domain adaptation strategy is employed to bridge the domain gap between batches with varying probability distributions, improving the alignment of batches.

**Fig. 1:**
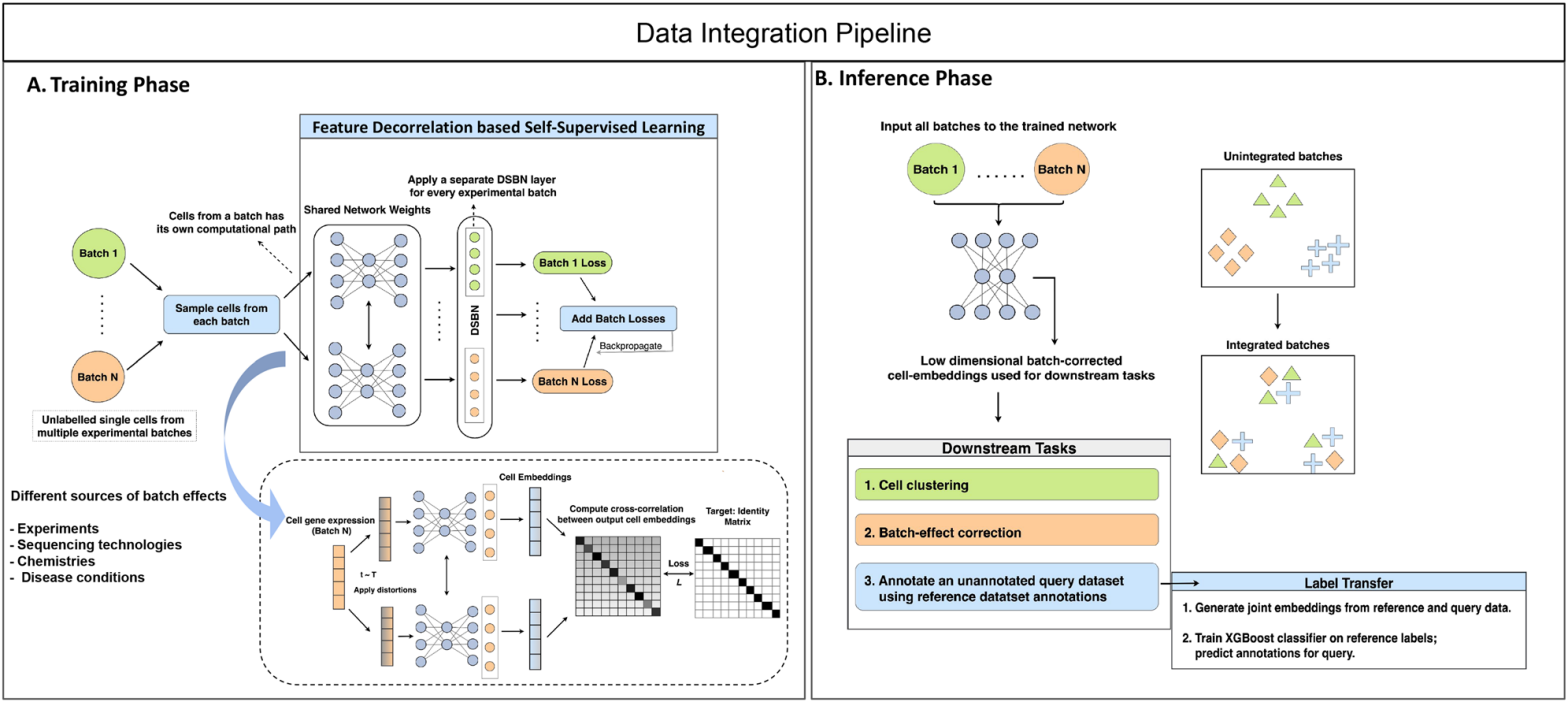
Overview of the scDecorr workflow - scDecorr takes as input single-cell gene-expression matrix coming from different studies (Domains) and uses a self-supervised feature decorrelation approach using a siamese twin model to obtain an optimal data representation. This representation is then used to perform typical single-cell downstream tasks such as clustering, batch-effect correction and cell-type annotation. We show the utility of scDecorr on 4 datasets from different tissues and technologies and compare it to other computational tools on similar tasks.

Here, we adopt these ideas and propose a novel *Feature Decorrelation based Representation Learning with Domain Adaptation* framework for integrative single-cell analysis. The key contributions of this paper are: 1. Learn robust cell representations of unlabelled single cell experiments in a negative-sample free self-supervised fashion, by leveraging the idea of feature decorrelation 2. Model the data integration problem using implicit domain adaptation 3. Extend feature decorrelation based SSL to an unsupervised domain adaptation context, where batch-invariant representations are learned from unannotated samples belonging to multiple domains (or batches) with different distributions 4. Comprehensive experimentation and benchmarking against state-of-the-art methods on diverse, standard scRNA-seq datasets exhibiting a range of batch effectsv(Fig. 1).

## Methods

### Overview

scDecorr employs self-supervised feature decorrelation–based representation learning^30^ to integrate multiple unlabeled single-cell datasets. The method learns representations by constructing two distorted views of each gene expression profile and passing them through a Siamese network with shared parameters. The training objective maximizes agreement between corresponding embedding dimensions across the two views while penalizing correlations between different dimensions, thereby encouraging informative yet non-redundant features.

To integrate data across domains, scDecorr uses a shared encoder for all batches while accounting for domain-specific differences through domain-specific batch normalization (DSBN)^33^. For each domain, embeddings are standardized so that each dimension has zero mean and unit variance within that domain. The same decorrelation objective is then applied independently to each domain. Because all domains share the same encoder and are optimized toward the same normalization and decorrelation targets (i.e., high agreement across views and low correlation between different embedding dimensions), the encoder is constrained to produce representations that follow the same structure across batches. As a result, cells from different domains are mapped into a common coordinate system, even though no explicit cross-domain matching loss is used. In this way, alignment is achieved implicitly: cells with similar biological profiles are positioned close to one another in the learned space, regardless of their batch of origin.

### Description

**Representation Learning: ** Representation learning using feature decorrelation involves using a Siamese deep network $f_{\theta }$ that projects samples from the original feature space (X) to embedding space $(Z_{\theta })$ via a joint embedding of distorted samples (Y). The network is trained with an objective such that the embeddings preserve as much information as possible from the presented samples while also achieving a high degree of invariance to the distortions applied^30^. This is achieved by maximising the mutual information $I(Z_{\theta };X)$ between the embeddings ($Z_{\theta }$) and presented samples (X), while minimising the mutual information $I(Z_{\theta };Y)$ between the embeddings ($Z_{\theta }$) and the distorted samples (Y). This can be briefly described using an objective function $L({\theta }):=I(Z_{\theta };Y)-I(Z_{\theta };X)$. The loss can be simplified and re-written as follows $L({\theta }):=H(Z_{\theta }|X)-\lambda H(Z_{\theta })$, where H(.) denotes the entropy and $\lambda$ is a positive constant. This simplified objective reveals that the conditional entropy $Z_{\theta }|X$ term needs to be minimized that is, $Z_{\theta }$ has to be completely determined from *X*. In other words, the information $Z_{\theta }$ contains about the distortions has to be minimized therefore, minimizing the conditional entropy loss term is equivalent to maximizing the alignment (or, similarity) between the embeddings of the distorted samples *Y*. As this loss term is essentially responsible for making the embeddings invariant to distortions applied, it is also called the *invariance term*. Furthermore, minimizing the overall objective results in maximizing the second loss term $H(Z_{\theta })$. Maximizing the entropy is aimed at enhancing the variability of the learned embeddings $Z_{\theta }$. This can be realized by minimizing the redundancy of information encoded by different vector components. To this end, the correlation between every pair of embedding components are reduced towards zero, which is commonly referred to as decorrelation. The rationale behind decorrelation is to avert the occurrence of an informational collapse, wherein components may become highly correlated or vary together. The second loss term is formally designated as the *decorrelation term*.

The joint embeddings of two distorted profiles of each cell $(Z,Z')$ is leveraged to calculate the invariance and covariance loss terms. Specifically, the cross-correlation matrix (W) between the embeddings is computed over a mini-batch as follows:

1 $$\begin{aligned} \mathcal {W}_{i,j}= \frac{1}{N_{b}}\frac{\sum _{b}(Z_{b,i}-\mu _{i})(Z'_{b,j}-\mu '_{j})}{\sigma (Z_{i})\sigma (Z'_{j})} \end{aligned}$$

where,

2 $$\begin{aligned} \mu _{i}=\frac{1}{N_{b}}\sum _{b}(Z_{b,i}); \mu '_{j}=\frac{1}{N_{b}}\sum _{b}(Z'_{b,j}) \end{aligned}$$

3 $$\begin{aligned} \sigma ^{2}(Z_{i})=\frac{1}{N_{b}}\sum _{b}(Z_{b,i}-\mu _{i})^2; \sigma ^{2}(Z'_{j})=\frac{1}{N_{b}}\sum _{b}(Z'_{b,j}-\mu '_{j})^2 \end{aligned}$$

where, *b* indicates the mini-batch index, $N_{b}$ indicates the size of the mini-batch, and i, j index the vector dimensions of the output embeddings.. $\mathcal {W}_{i,j}$ values are comprised between 1 (perfect correlation) to −1 (anti-correlation), and 0 indictating uncorrelatedness.

The *invariance term* of the loss is computed using the on-diagonal elements of $\mathcal {W}$, encouraging them to be close to 1

4 $$\begin{aligned} \mathcal {L}_{invar}=\sum _{i}(1-\mathcal {W}_{ii})^2 \end{aligned}$$

whereas the *decorrelation term* of the loss is calculated using the off-diagonal elements, encouraging them to be close to 0

5 $$\begin{aligned} \mathcal {L}_{decorr}=\sum _i \sum _{j \ne i} \mathcal {W}_{i j} ^2 \end{aligned}$$

The overall loss is therefore,

6 $$\begin{aligned} \mathcal {L} = \mathcal {L}_{invar} + \lambda \mathcal {L}_{decorr} \end{aligned}$$

where $\lambda$ is a trade-off factor balancing the invariance and decorrelation terms.

**Domain Adaptation: **However, when a mini-batch comprises of cell samples from different domains, the usual computation of the loss terms results in erroneous results. This issue arises because $\mathcal {W}$, $\mu$, and $\sigma$ are calculated over the mini-batch. Since cell samples from different domains follow distinct probability distributions, they possess varying mean and variance values. Therefore, computing a single mean $\mu$ and variance $\sigma$ for all the domains hurts the domain alignment performance. To address the challenge of domain differences in a mini-batch, we adopt a strategy where separate values of $\mathcal {W}^{(d)}$, $\mu ^{(d)}$, and $\sigma ^{(d)}$ are calculated independently for each domain *d*. This is achieved through random sampling of cells from each domain, and normalizing the data independently across domain via domain-specific batch normalization layers^33^. Therefore, the loss term in Eq 6$\mathcal {L}^{(d)}$ is computed independently for every domain and the overall domain adapted loss $L_{DA}$ is simply the mean of all the domain losses.

7 $$\begin{aligned} \mathcal {L}_{DA} = \frac{1}{D}\sum _{d}\mathcal {L}^{(d)} \end{aligned}$$

### Method implementation for single-cell transcriptomics data

#### Preprocessing

First, we remove genes expressed in less than 3 cells. Next, we log-normalize the raw gene expression counts data using Scanpy’s^34^ “normalize_total” function with a target sum of 1*e*4. Finally, we select the top 2000 highly variable genes (HVG) by batch for training scDecorr. The HVGs were selected using the “Seuratv3” flavor of Scanpy^34^ HVG selection module.

#### scDecorr workflow

scDecorr accepts *D* preprocessed gene expression count matrices $\mathcal {X}=\{\mathcal {X}^{(1)},...,\mathcal {X}^{(D)}\}$ from *D* distinct domains (or, sources, or batches) as input. scDecorr aims to integrate these *D* unintegrated count matrices to a common domain-invariant low-dimensional representation space $\mathcal {Z}$. In our experiments, the input space has a size of 2000 corresponding to the highly variable genes and the representation space has a size of 64.

**Mini-Batch Construction:** First, the mini-batch is constructed by randomly sampling *K* cells from all domains. $\{X^{(d)} \subseteq \mathcal {X}^{(d)} s.t. \Vert X^{(d)}\Vert = K\}$ constitute the mini-batch specific to the domain.

**Data Augmentation:** Next, for each domain specific mini-batch $X^{(d)}$, mini-batches of two distorted views $X'^{(d)}$ and $X''^{(d)}$ are created by sampling two transformations from a distribution of data augmentations. The transformations include randomly zeroing out $20\%$ of genes, and randomly shuffling $10\%$ of the gene expression values. Each individual transformation is applied with probability of 0.5. We also experiment with other augmentation schemes and empirically verify that this combination of transformations perform the best. These results are presented in Table S9.1 and Section 2.1 of the supplement.

**Model Architecture:** scDecorr has a symmetric Siamese net architecture— network consists of 2 identical branches, each responsible for embedding a particular distorted view. Each branch consists of an encoder and projector network, and their weights are shared between the branches. For every domain, the mini-batches of distorted views $X'^{(d)}$ and $X''^{(d)}$ are fed to the respective branches of scDecorr. The encoder network $f_{\theta }$ produces batches of low-dimensional representations $Z'^{(d)}=f_{\theta }(X'^{(d)})$ and $Z''^{(d)}=f_{\theta }(X''^{(d)})$. The representations are then fed to a projector network $g_{\phi }$ which projects the low-dimensional representations to a high dimensional embedding space, $Y'^{(d)}=g_{\phi }(Z'^{(d)})$ and $Y''^{(d)}=g_{\phi }(Z''^{(d)})$. The output of the encoder *Z* is called *representations* and the output of the projector *Y* is called *embeddings*. The encoder consists of a densely connected neural network^19,35^, specifically a dense multi-layer perceptron (MLP), which we will refer to as DenseNet throughout the paper. The projector consists of 3 linear layers, the first two being followed by a ReLU and a domain-specific batch normalization layer.

**Loss Computation:** Finally, the batches of embeddings of every domain $Y'^{(d)}$ and $Y''^{(d)}$ are z-score normalized (along the batch axis) independently across domains using another domain-specific batch-normalization layer with no affine transform. This ensures that every embedding component of a domain has a mean of 0 and variance of 1 over its mini-batch so that the domain specific cross-correlation matrix can be computed simply by a matrix multiplication between $Y'^{(d)}$ and $Y''^{(d)}$:

8 $$\begin{aligned} \mathcal {W}^{(d)} = \frac{1}{K} Y'^{(d) T} Y''^{(d)} \end{aligned}$$

The domain loss $\mathcal {L}^{(d)}$ is then computed from $\mathcal {W}^{(d)}$ as follows:

9 $$\begin{aligned} \mathcal {L}^{(d)} = \sum _{i}(1-\mathcal {W}^{(d)}_{ii})^{2} + \lambda \sum _{i} \sum _{j \ne i} \mathcal {W}^{(d) 2}_{i j} \end{aligned}$$

The overall loss can simply be computed as the average of all the domain losses.

10 $$\begin{aligned} \mathcal {L} = \frac{1}{D}(\sum _{d}\sum _{i}(1-\mathcal {W}^{(d)}_{ii})^2 + \lambda \sum _{d}\sum _{i} \sum _{j \ne i} \mathcal {W}^{(d) 2}_{i j}) \end{aligned}$$

where i, j index the vector dimensions of the output embeddings.

**Model Optimization:** We train scDecorr for 1000 epochs using the Adam optimizer with a base learning rate of 0.0003. Cosine annealing with a warm-up of 10 epochs is used to anneal the learning rate over time. Early stopping is also used to stop training if there is no improvement in validation loss for 50 epochs.

After training scDecorr on the unintegrated dataset $\mathcal {X}$, the integrated 64 dimensional representations $\mathcal {Z}=f(\mathcal {X})$ are used as features for downstream tasks.

**Training Configuration:** In our experiments, we use two different configurations *S* and *L* for training scDecorr. Configuration S is used for small datasets (with $\le 50k$ cells) and configuration L for large-scale datasets (with $\ge 50k$ cells). For configuration *S*, a mini-batch size of $K=512$ is used. Moreover, an 11 layer DenseNet model and a $512-512-512$ layer MLP are used as the encoder and decoder respectively. Furthermore, for configuration *L*, a mini-batch size of $K=2048$ is used. Moreover, a 21 layer DenseNet model^35^ and a $1024-1024-1024$ layer MLP are used as the encoder and decoder respectively. The best training configurations of scDecorr for all the benchmark datasets are presented in Table S2. We conduct systematic hyperparameter search to find the best training configurations, the results of which are presented in Table S9.2.

### Downstream evaluation

We evaluate the performance of scDecorr using two downstream tasks namely data integration and and reference to query label transfer. Moreover, we systematically benchmark the performance of scDecorr on these tasks with other contemporary state-of-the-art data integration methods. Due to stochasticity of deep learning models, each method is run with 5 individual seeds for fair comparison.

#### Data integration task

We evaluate the data integration performance of a method by assessing its ability to preserve biological signals, correct batch effects while simultaneously ensuring that batches are not overcorrected i.e. distinct cell-types from different batches are not mixed together. The evaluation metrics are computed using the scib package^6^, unless otherwise specified. The author provided cell-type annotations and batch labels are used for evaluation.

**Clustering (Biological conservation)** aims to check whether biologically similar cells can be grouped together in the cell representation space. This is assessed by measuring cell-type clustering performance on the batch-integrated representations obtained from each method. We run the Leiden clustering algorithm using resolutions evenly spaced from 0.1 to 1.0 with a stride of 0.1 and report the best average ARI and NMI score. This is done to ensure a fair comparison between all baseline methods, as the optimal clustering resolution might depend on the nature of their representations.

Three metrics, namely, the Adjusted Rand Index (ARI)^36^, Normalized Mutual Information (NMI)^6,37^, and cell-type silhouette (cSil) score^6,38^, are utilized to evaluate the clustering performance.

The Adjusted Rand Index (ARI)^36^ quantifies the extent of agreement between the clustering labels and the ground truth cell-type labels, considering the agreement beyond what would be expected by chance. A higher ARI value, ranging from −1 to 1, indicates a better agreement between the predicted clusters and the true labels, with 1 indicating a perfect match.

The Normalized Mutual Information (NMI)^6,37^ measures the mutual information between the predicted clusters and the true labels, normalized by the entropy of the predicted and true label distributions. NMI values range from 0 to 1, with 1 indicating a perfect match between the predicted clusters and the true labels.

The cell-type average silhouette width (cASW)^6,38^ evaluates the degree of separation between the clusters. It assesses how well each cell is assigned to its corresponding cluster compared to other clusters. The silhouette score ranges from 0 to 1, with higher values indicating better separation between clusters and overall improved clustering performance. A score close to 1 implies well-separated and distinct clusters, while a score close to 0 suggests overlapping or poorly separated clusters.

These 3 scores are finally aggregated into a $S_{bio}$ score as described later.

**Batch-effect correction** is assessed using several measures such as Batch Average Silhouette Width (Batch ASW)^39^, Graph Connectivity^6^, and Batch Entropy^40^ on the integrated representation space.

The average silhouette width with batch labels (bASW)^39^ measures batch mixing by determining the silhouette width for each cell type using batch labels and then averaging across all cell types. A value of 1 indicates an ideal mixing scenario, while 0 suggests strong separation between batches.

Graph connectivity measures how well connected are the cells of a certain cell-type in the nearest neighborhood graph. For each cell identity label, a subgraph is constructed containing only cells of that label, and the fraction of cells belonging to the largest connected component is computed. The final score is obtained by averaging this fraction across all cell identities. Graph connectivity values range from 0 to 1, where a score of 1 indicates that all cells of each identity are fully connected in the kNN graph. The kNN graph is constructed using the integrated representation space.

Batch entropy mixing score measures batch integration by computing the regional mixing entropy of cells from different batches. A high score implies that cells from different batches are well mixed together.

It is to be noted that batch ASW and batch entropy mixing scores are only computed on cell-types shared across multiple batches. Finally, these 3 scores are aggregated into a $S_{batch}$ score as described later.

**Overcorrection** is a general issue in scRNA-seq data integration methods. Some methods aggressively correct batch effects which results in distinct cell-types from different batches getting mixed together. We follow the implementation of the overcorrection score from SCALEX^40^ to quantify this. The authors originally implement it as a negative index i.e. higher overcorrection score implies greater degree of distinct cell-types mixing. We convert this to a positive index to ensure parity with other metrics during benchmarking.

**Score Aggregation:** Following^6^, score aggregation is performed to compute an overall performance measure for each subtask such as $S_{bio}$ or $S_{batch}$. To ensure that each individual score (eg. NMI, ARI, cASW) has equal weightage in the aggregated score ($S_{bio}$), each individual score is first min-max normalised (with a range between 0 to 1) across all method runs within a dataset. An overall score for that subtask is then simply a mean of the normalised individual scores. This ensures comparable overall performance scores and allows for ranking methods both within and across datasets. The aggregated scores are formally defined as follows:

11 $$\begin{aligned} S_{bio} = \frac{f(ARI)+f(NMI)+f(cASW)}{3} \end{aligned}$$

12 $$\begin{aligned} S_{batch} = \frac{f(bASW) + f(Graph Connect) + f(Batch Entropy)}{3} \end{aligned}$$

13 $$\begin{aligned} S_{oc} = f(Overcorrection) \end{aligned}$$

where, *f*() is a min-max normalizing function which normalizes raw individual scores across all method runs within a dataset.

The overall data integration performance of a benchmark method on a scRNA-seq dataset is evaluated using,

14 $$\begin{aligned} S_{data\_integration}=0.6*S_{bio}+0.3*S_{batch}+0.1*S_{oc} \end{aligned}$$

The aggregation for integration score assigns a higher weight to the clustering (biological conservation) scores, highlighting the greater significance of preserving biological information compared to batch mixing^5,6^. Moreover, since overcorrection is an important factor in understanding data integration quality, the overcorrection score is included to the overall data integration score with a small weight.

#### Label transferring task

The process of label transferring involves assigning cell-type labels to an unlabeled query dataset using information from a reference dataset. In the case of scRNA-seq data, where gene expression data originates from multiple sources such as different platforms, datasets, or batches, we evaluate the performance of benchmark methods in annotating cell types across different sources. To accomplish this, we treat cells from a particular source as the query dataset, while considering the rest of the cells as the reference dataset.

Assuming that the data has already been integrated using a specific method, we evaluate the label annotation performance through the following approaches:

**Clustering (Biological Conservation) of Query:** Initially, we apply the Leiden clustering algorithm to the batch-integrated representations of the entire dataset. This enables us to evaluate the biological conservation quality of the query cells. We compute metric scores such as ARI (Adjusted Rand Index), NMI (Normalized Mutual Information), and cell-type ASW (cell-type silhouette) by comparing the cluster labels assigned by Leiden clustering to the ground truth cell-type labels of the query cells. This evaluation strategy is particularly valuable for datasets with minimal shared cell types between the reference and query datasets. The overall clustering performance of a method on the query cells is determined using the $S_{bw\_bio}$ score as described below.

**Classification of Query:** Next, we fit an K-nearest neighbors classifier model using the features of the reference cells and evaluate its performance on the query cells. Only the common cell types between the reference and query datasets are used for fitting and evaluation. The label annotation classification performance is assessed using metrics such as balanced accuracy and macro F1 scores. This evaluation strategy is particularly useful for datasets with a substantial number of shared cell types between the reference and query datasets. The overall classification performance of a method on the query cells is computed using the $S_{classify}$ score as described below. Before evaluation on a query dataset, we first select the optimal number of neighbors by running 3-fold grid search cross-validation on the reference data across *k* ranging from 5 to 200 with increments of 6. This is done so as to ensure fair comparison between all benchmark methods.

In summary, we evaluate the inter-batch label annotation performance of benchmark methods by assessing their performance in clustering and classifying the query cells using reference information.

**Score Aggregation:** The individual clustering and classification scores on a batch are aggregated in a similar fashion as described in Data integration task. Additionally, we now normalize the individual metrics scores across all methods runs only within each batch of a dataset. The normalised individual scores are then aggregated to compute batch-wise bio conservation score $S_{bw\_bio}$ and classification score $S_{classify}$ for each batch *b* as follows:

15 $$\begin{aligned} S^{b}_{bw\_bio} = \frac{f_b(ARI^b)+f_b(NMI^b)+f_b(cASW^b)}{3} \end{aligned}$$

16 $$\begin{aligned} S^{b}_{classify} = \frac{f_b(F1^b)+f_b(Acc^b)}{2} \end{aligned}$$

where, $f_b()$ is a min-max normalizing function which normalizes raw individual scores across all method runs within batch *b* of a dataset.

The overall label transfer performance of a method on a query batch is evaluated using:

17 $$\begin{aligned} S^b_{label\_transfer}=0.5*S^{b}_{classify}+0.5*S^{b}_{bw\_bio} \end{aligned}$$

The batchwise scores are finally aggregated to compute overall label transfer performance over the entire dataset as follows:

18 $$\begin{aligned} S_{bw\_bio}=\frac{1}{|B|}\sum _{b \in B}S^{b}_{bw\_bio} \end{aligned}$$

19 $$\begin{aligned} S_{classify}=\frac{1}{|B|}\sum _{b \in B}S^{b}_{classify} \end{aligned}$$

20 $$\begin{aligned} S_{label\_transfer}=\frac{1}{|B|}\sum _{b \in B}S^b_{label\_transfer} \end{aligned}$$

where B is the set of all batches in a dataset.

#### Ranking benchmark methods

**Fig. 2:**
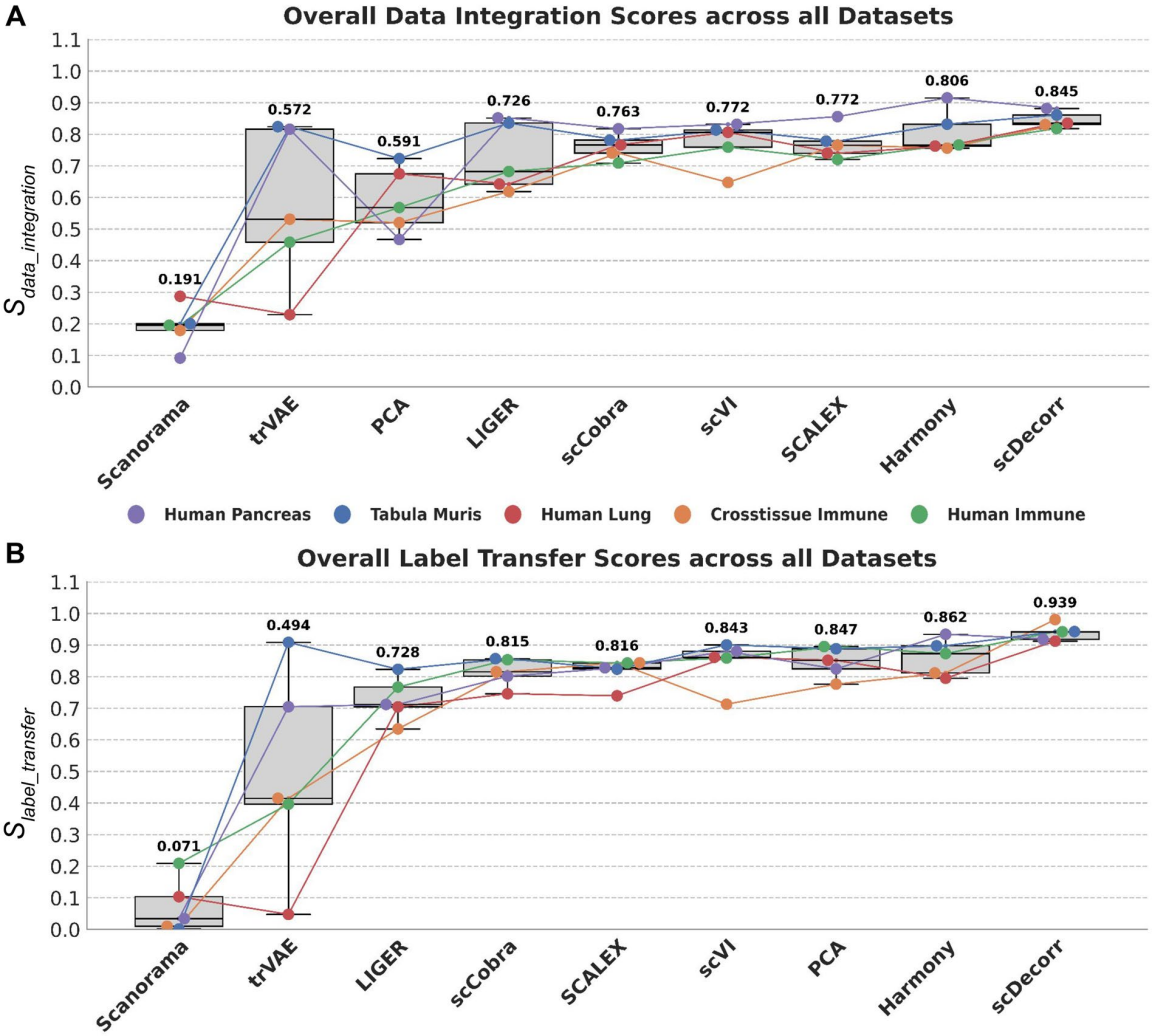
Ranking of benchmark methods according to overall A) data integration ($S_{data\_integration}$) and B) label transfer performance ($S_{label\_transfer}$) across 5 independent datasets namely crosstissue immune, human immune, human lung, human pancreas, and tabula muris. Scores from multiple runs were averaged before plotting. The mean score across datasets is annotated over each box in bold. scDecorr outperforms benchmark methods in both tasks.

Finally, using the aggregated scores from each task such as $S_{data\_integration}$ and $S_{label\_transfer}$, we rank all benchmark methods across datasets. This helps to systematically judge the overall performance of scDecorr in comparison to other benchmark methods. Scores from multiple seed runs are aggregated before ranking. The rankings are displayed in Fig. 2

### Data visualization

To visualize the data integration process, we employ UMAP embeddings^41^ of the batch-integrated feature representations, generated by a specific method. The label annotation UMAP plot of the complete dataset is constructed by aggregating the predicted cell-type labels from each query batch. These labels are obtained using a leave-one-out label transfer scheme (Refer to Label Annotation in Section Label transferring task). For visualizing metric scores, we use bar-plots with error bars to denote the variability between runs. To visualize overall performance of benchmark methods across datasets, we use annotated boxplots and order the methods from lowest to highest scores (See Fig. 2).

## Results

We benchmark scDecorr on 5 diverse public scRNA-seq datasets namely, Human Lung^6^, Human Immune^6^, Cross-tissue Immune^42^, Human Pancreas^6^, and Tabula Muris^3^. These datasets encompass a range of data integration tasks such as integration across donors, sequencing technologies, platforms etc. They also encompass diverse tissues, organs, cell-types and species. The cell type annotations and batch labels of these datasets are available from original publications. However, the cell-type labels are not required for training scDecorr and are only used during downstream evaluation. An overview of the benchmark datasets is presented below (Table 1).

**Table 1:** Datasets details - overview of datasets used in this study.

Dataset	# Batches (Integration)	# Batches (Label Transfer)	# Cells	# Cell Types	Shared Cell Types (%) ($\ge 2$ batches)	Shared Cell Types (%) (All batches)
Crosstissue Immune^42^	3	3	216611	18	100	100
Human Immune^6^	10	5	33506	16	75	19
Human Lung^6^	16	3	32472	17	65	53
Human Pancreas^6^	9	6	16382	14	93	29
Tabula Muris^3^	2	2	67354	28	93	93

1. Data integration is performed on individual donor batches.

2. Label transfer is performed on distinct experimental batches (group of donor batches).

3. Shared cell types are computed on the label transfer batches.

For other relevant detials such as batch names, please refer to Table S1.

### Experimental setup

**Fig. 3:**
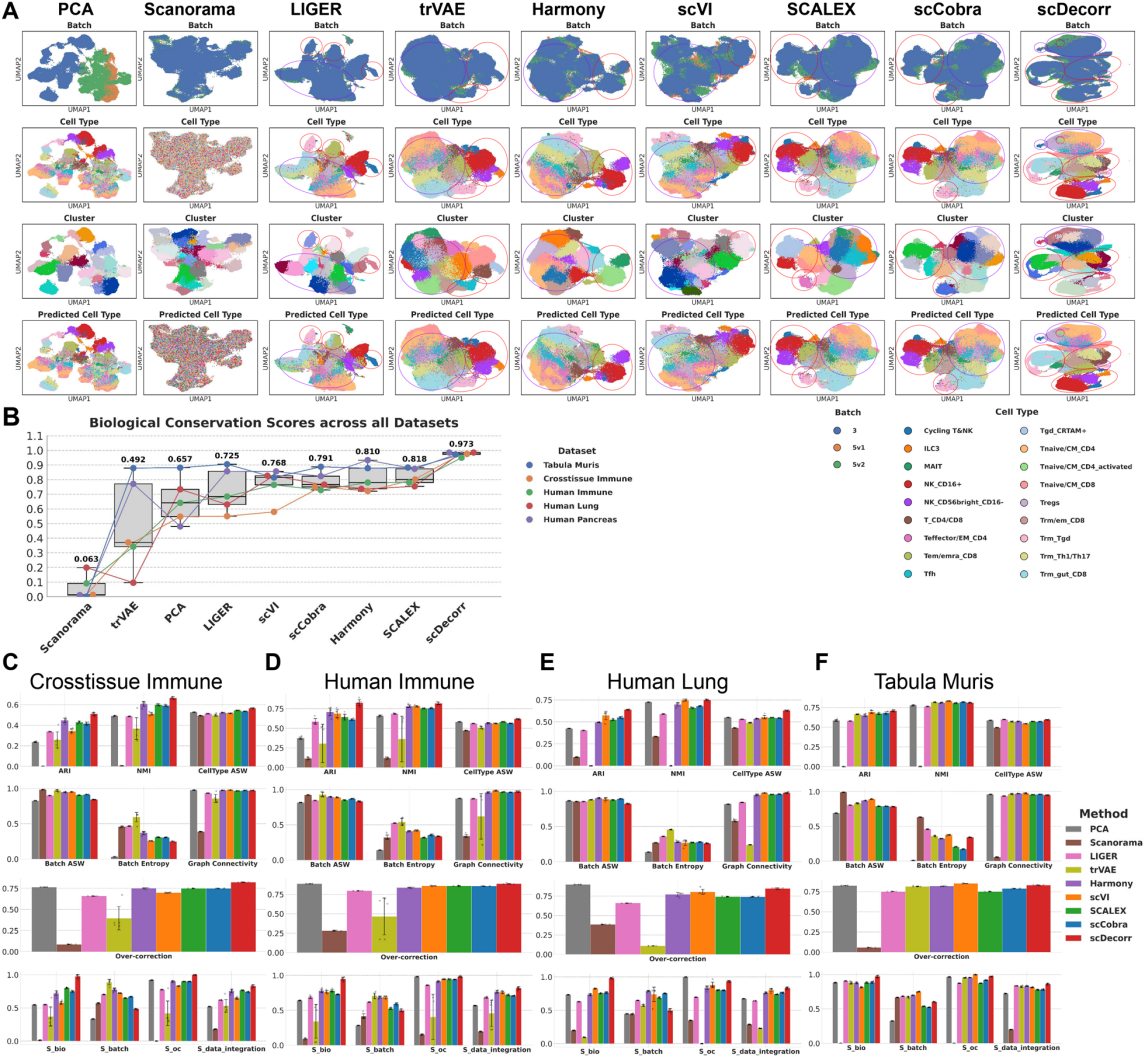
Data integration performance of benchmark methods. A. UMAP plots of embeddings of benchmark methods on cross-tissue immune atlas, B. Boxplots denoting biological conservation performance ($S_{bio}$) of methods on all benchmarking datasets, C,D,E,F. Barplots indicating data integration scores of benchmark methods on cross-tissue immune atlas, human immune, human lung and tabula muris datasets respectively.

To evaluate the performance of scDecorr, we assess its effectiveness in two different downstream scenarios: data integration and label transfer. To establish a benchmark, we compare scDecorr with 7 other methods: Harmony^10^, scVI^12^, scCobra^43^, SCALEX^40^, trVAE^44^, LIGER^45^, Scanorama^46^. For a baseline performance of raw unintegrated data, we also compare our results with standard PCA^47^ embeddings. To ensure fairness, all methods were evaluated using identical preprocessing and downstream evaluation; methods requiring distinct preprocessing pipelines such as Seurat^48^ were excluded. For our experiments, we utilize the default implementations of these methods available in the scib package^6^. For PCA and Harmony, we use the Scanpy^34^ framework. SCALEX and scCobra were run using their respective github packages. For generating LIGER representations, we employ the default implementation from the pyLIGER package^49^.Due to stochasticity of a few methods in the benchmark, each method is run with 5 random seeds for fair comparison. Subsequently, the batch-integrated feature representations extracted by these methods serve as the input for the downstream tasks. To enable fair comparison between benchmark methods, input dimensions of all methods were kept fixed to 2000.

For the data integration experiments, we integrate the individual donor batches in a dataset (data integration batches presented in Table S1) and evaluate methods on the same. For our label transfer experiments, we treat distinct experimental sources or sequencing technologies as the query datasets and not the individual batches. (Details of label transfer batches shown in Table S1). This is done because usually batches correspond to individual donors in an experiment. Instead we treat high level batches (distinct experimental sources) as our query datasets which aligns more towards the idea of label transfer across different experiments and also which is a more challenging task than label transfer across donor batches.

Next, we discuss the benchmarking results and also discuss the advantages and potential limitations of scDecorr.

### scDecorr outperforms benchmark methods in biological conservation

We systematically benchmarked the data integration performance of scDecorr on multiple complex and heterogeneous datasets, including the large Cross-tissue Immune Atlas, and a mouse atlas dataset. Together, these datasets span diverse biological systems, sequencing technologies, and batch structures, providing a rigorous evaluation of integration performance. In this section, we demonstrate that scDecorr consistently integrates these datasets while achieving superior biological conservation.

Across all evaluated datasets, scDecorr achieves the strongest biological conservation performance compared to all benchmark methods (Fig. 3B). Specifically, scDecorr attains a mean $S_{bio}$ score of 0.973 across datasets, representing an improvement of nearly $19\%$ over the next best-performing method, SCALEX^40^, which achieves a mean $S_{bio}$ score of 0.818. Beyond its high average performance, scDecorr exhibits remarkably consistent biological conservation across datasets, as reflected by the low variance in its $S_{bio}$ scores (Fig. 3B). In contrast, most competing methods display substantial variability in biological conservation depending on dataset characteristics.

**Fig. 4:**
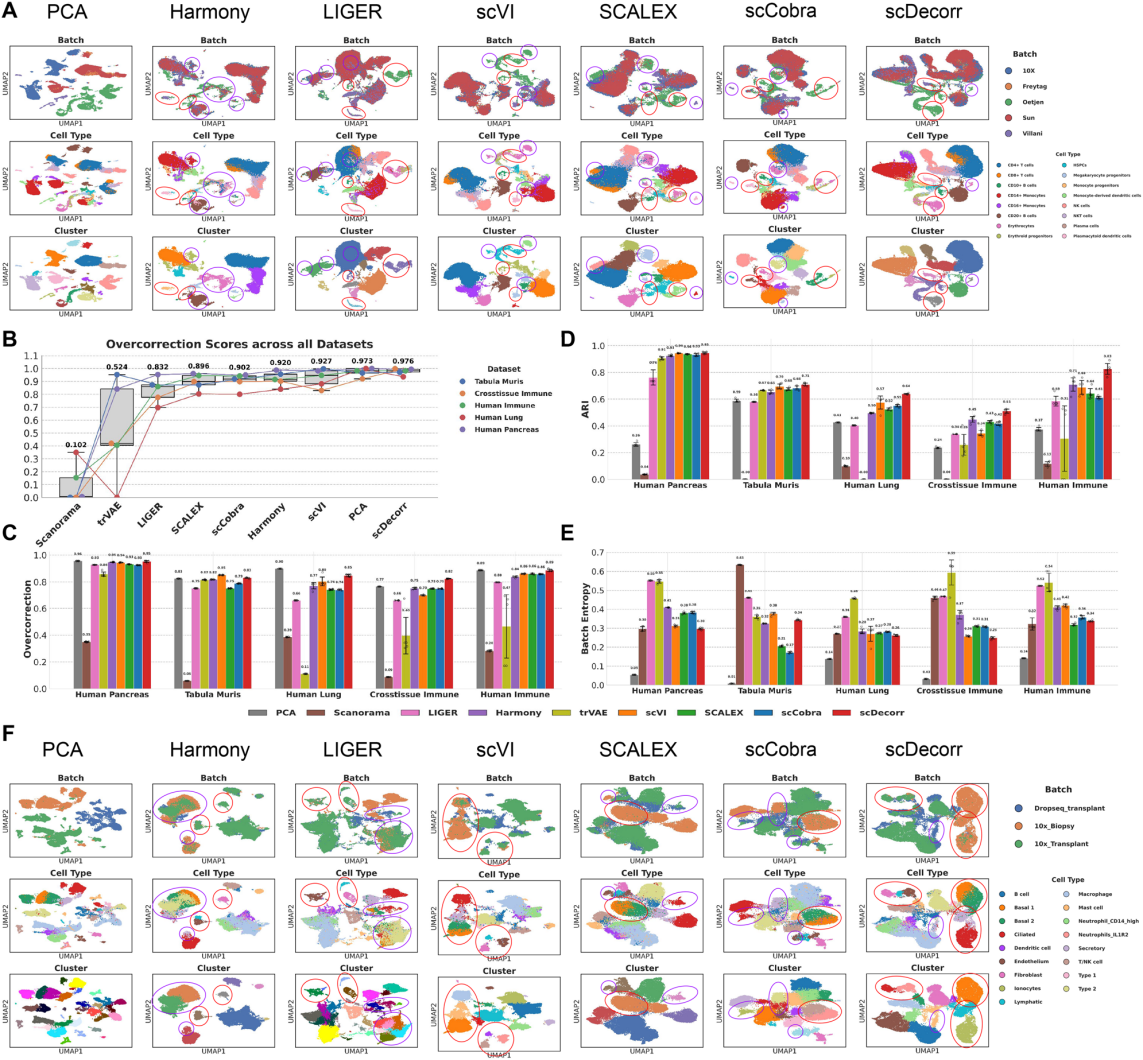
Overcorrection performance of benchmark methods. A) UMAPs of benchmark methods on human immune. Highlighted cell types include Plasmacytoid dendritic cells, a rare cell-type and batch-specific cell-types of Oetjen study such as Erythrocytes, and CD10+ B cells. UMAP regions where methods succeed are highlighted in red, Regions where methods fail are highlighted in violet. B) Boxplots denoting overcorrection performance ($S_{oc}$) of methods on all benchmarking datasets, C) Barplots indicating raw overcorrection scores of methods across all benchmark datasets, D) Barplots indicating raw ARI scores of methods across all benchmark datasets, E) Barplots indicating raw batch entropy scores of methods across all benchmark datasets, F) UMAPs of benchmark methods on human immune. Highlighted cell types include Oetjen study specific cell-types such as Basal-1, Basal-2, major cell-types such as Secretory, Ciliated, rare cell-type such as Lymphatic and other cell-types such as Fibroblast, Endothelium cells. UMAP regions where methods succeed are highlighted in red, Regions where methods fail are highlighted in violet.

Consistent with these findings, scDecorr also achieves higher ARI scores than other methods across all datasets, indicating more accurate recovery of cell-type structure following integration (Fig. 4D). Furthermore, when considering overall data integration performance across all evaluation metrics, scDecorr ranks highest among all benchmarked methods (Fig. 2), followed by Harmony and SCALEX.

Collectively, these results demonstrate that scDecorr provides consistently strong and robust biological conservation across diverse integration scenarios, establishing it as a reliable and effective data integration method. Next, we present a detailed analysis of the Cross-tissue Immune Atlas and Tabula Muris datasets.

**Cross-tissue Immune Atlas**: We evaluated scDecorr on the cross-tissue immune cell atlas dataset^42^, which comprises over 200,000 cells sequenced from 12 adult donors across 17 organs and represents a challenging data integration setting. We focused on the *T and innate lymphoid cells* subset and assessed integration across sequencing chemistries. This subset contains 216,611 cells sampled from 17 organs, including lung, lymph nodes, liver, and spleen, and profiled using three 10x Genomics chemistries: 5’ v1, 5’ v2, and 3’, consisting of 41,715, 61,559, and 113,337 cells, respectively. The dataset includes 18 distinct cell types, all of which are represented across all three chemistries. Strong batch effects are evident in the unintegrated PCA-based UMAP (Fig. 3A), motivating the need for effective integration across chemistries.

Figure 3C summarizes the quantitative integration performance of all benchmarked methods on this dataset. scDecorr achieves the highest biological conservation score ($S_{bio}$), outperforming all competing methods by a substantial margin. Specifically, scDecorr improves upon the next best-performing methods–SCALEX, scCobra, and Harmony–by $22\%$, $30\%$, and $35\%$, respectively, attaining an average $S_{bio}$ score of 0.98 compared to 0.80, 0.75, and 0.72 for SCALEX, scCobra, and Harmony (Table S5.1) respectively. Relative to these methods, scDecorr also demonstrates consistent improvements of $13\%-21\%$ in ARI, $10\%-13\%$ in NMI, and $4\%-10\%$ in cell-type silhouette scores (Table S4.1, Figure S4). Other methods, including scVI, LIGER, trVAE, and PCA, show weaker biological conservation, with LIGER and PCA achieving comparable performance. Biological conservation scores are generally stable across random seeds for all methods except trVAE, which exhibits substantial variance.

scDecorr also demonstrates competitive batch correction performance, achieving an average batch entropy score of 0.25 (Fig 4E, Table S4.1) a substantial improvement over unintegrated PCA embeddings, which attain a score of only 0.03. The batch entropy score achieved by scDecorr is comparable to that of SCALEX (0.31), scCobra (0.31), and scVI (0.26). trVAE achieves the highest batch entropy score (0.59) in this dataset, however, trVAE’s superior batch mixing (0.59) comes at the cost of severe overcorrection, as reflected by its low overcorrection score (0.40) and poor biological conservation. In contrast, scDecorr minimizes overcorrection and achieves the highest overcorrection score (0.82) among all methods (Fig 4C, Table S4.1). Moreover, scDecorr achieves the best overall data integration performance ($S_{data\_integration}$) outperforming the next best method SCALEX by approximately $9\%$ (Table S5.1). Taken together, these results indicate that scDecorr provides the best overall integration performance by balancing batch correction and biological preservation.

Strong batch correction alone does not guarantee high-quality data integration, as there is an inherent trade-off between batch mixing and biological conservation. This trade-off is clearly illustrated in the UMAP visualizations (Fig. 3A). trVAE achieves near-perfect batch mixing but collapses many biologically distinct populations, resulting in poorly separated clusters. In particular, trVAE incorrectly merges Tnaive/CM_CD4 with Tnaive/CM_CD8 cells; Trm_gut_CD8 with Trm_Th1/Th17, Trm/em_CD8, and partially Trm_Tgd cells. Although it distinguishes NK_CD16+ from NK_CD56bright_CD16- cells, the resulting clusters are not well separated.

In contrast, scDecorr produces well-defined and biologically meaningful clusters. It clearly separates NK_CD16+, NK_CD56bright_CD16-, ILC3, and Cyclic T&NK cell types, as shown in both the cell-type and cluster UMAPs (Fig. 3A). scDecorr also distinctly resolves Trm_gut_CD8, Trm_Tgd, Trm_Th1/Th17, and Trm/em_CD8 populations, forming well-separated clusters that are accurately identified by the Leiden algorithm. A small fraction of Trm_Tgd cells appears disconnected from the main cluster (marked in violet in Fig. 3A), but these cells are still correctly assigned to the same cluster by Leiden.

scDecorr further distinguishes Tnaive/CM_CD4 from Tnaive/CM_CD8 cells, a task at which most competing methods fail. Although Tnaive/CM_CD4 clusters partially overlap with Teffector/EM_CD4, Tfh, and Tregs in the UMAP, Leiden clustering is still able to identify these populations as roughly distinct clusters (Fig. 3A). scDecorr also successfully detects Tregs and T_CD4/CD8 populations that are missed or poorly resolved by many other methods.

Importantly, rare cell types ($\le 5{,}000$ cells), including ILC3 (1,312 cells), MAIT (4,849 cells), and Cyclic T&NK (2,126 cells), are well preserved and not absorbed into larger clusters. For Cyclic T&NK cells, while the majority of cells form a distinct cluster, a small subset is dispersed throughout the embedding (marked in violet in Fig. 3A). Tnaive/CM_CD4_activated cells (3,748 cells) are partially merged with Tnaive/CM_CD4 and Tregs in the UMAP, but Leiden successfully identifies them as a separate cluster (marked in red in Fig. 3A). Additionally, a small fraction of Tgd_CRTAM+ cells (4,690 cells) is merged with Tem/emra_CD8 and Trm/em_CD8 cells near cluster boundaries.

scCobra successfully clusters NK_CD16+, NK_CD56bright_CD16-, ILC3, Tem/emra_CD8, and Trm_Tgd populations, but the resulting cluster boundaries are poorly defined. Many other cell types–including Tnaive/CM_CD4, Tnaive/CM_CD8, Tnaive/CM_CD4_activated, Teffector/EM_CD4, Tfh, Tregs, and MAIT–are heavily mixed. scCobra also fails to clearly resolve Trm cell states, particularly Trm/em_CD8, Trm_gut_CD8, and Trm_Th1/Th17, and completely merges the Tgd_CRTAM+ cluster with Trm/em_CD8.

The qualitative performance of SCALEX is largely similar to scCobra, with the additional issue that Tem/emra_CD8 cells partially merge with NK_CD16+ cells, and a large fraction of Trm_Tgd cells merge with Trm_gut_CD8. Otherwise, the UMAP structures produced by scCobra and SCALEX are highly similar.

scVI merges most distinct cell types and fails to produce well-separated clusters. NK_CD16+ and NK_CD56bright_CD16- populations are not clearly separated and mix with other cell types near cluster boundaries. Additionally, many cell-type clusters are fragmented and scattered throughout the UMAP space.

Harmony successfully separates NK_CD16+ and NK_CD56bright_CD16- populations; however, a substantial fraction of NK_CD56bright_CD16- cells merges with NK_CD16+ cells. Moreover, a large portion of Cyclic T&NK cells is merged with NK_CD16+ cells. Harmony fails to distinguish Tnaive cell states, merging Tnaive/CM_CD4, Tnaive/CM_CD8, and Tnaive/CM_CD4_activated populations, and does not adequately resolve Trm cell states, particularly Trm/em_CD8, Trm_gut_CD8, and Trm_Th1/Th17.

LIGER successfully clusters Trm_Tgd, Tem/emra_CD8, and Cyclic T&NK populations but performs poorly on most other cell types. Scanorama exhibits extensive mixing across all cell types, resulting in very poor biological conservation performance.

**Tabula Muris**: We evaluated scDecorr on the Tabula Muris atlas^3^, which comprises 67, 354 single cells profiled across 24 mouse organs and includes 28 distinct cell types, making it a challenging and complex data integration task. The dataset was generated using two sequencing technologies: Droplet (47, 664 cells) and FACS (19, 690 cells). Most cell types are shared between the two technologies, with the exception of kidney proximal convoluted tubule epithelial cells, which are exclusive to the Droplet batch, and microglial cells, which are exclusive to the FACS batch. PCA-based UMAP visualization (Fig S11A) reveals a strong batch effect between the two technologies, motivating the need for integration between technologies.

As shown in Figure S14B and Table S5.1, scDecorr achieves the best biological conservation and overall data integration performance among all benchmarked methods. Specifically, scDecorr attains a mean $S_{bio}$ score of 0.972 and a mean $S_{data\_integration}$ score of 0.861, representing improvements of $7.4\%$ and $3\%$ respectively, over the next best-performing method, LIGER, which achieves $S_{bio}$ and $S_{data\_integration}$ scores of 0.905 and 0.835 (Table S5.1). While scDecorr does not achieve the highest mean batch-correction score ($S_{batch} = 0.602$), its performance exceeds that of scCobra (0.523), SCALEX (0.538), and unintegrated PCA (0.325). In addition, scDecorr also achieves competitive overcorrection scores–attaining a mean overcorrection score ($S_{oc}$) of 0.971, which is higher than all methods except scVI, which achieves a score of 0.997.

We next perform a qualitative assessment of the integration results. Figure S14A presents the UMAP visualizations for all methods on the Tabula Muris dataset. scDecorr distinctly clusters B cells, the largest cell population in the dataset. It also clearly separates hepatocyte, bladder urothelial cell, bronchial smooth muscle cell, basal cell, skeletal muscle satellite cell, and microglial cell types. Importantly, scDecorr preserves batch-specific cell types–kidney proximal convoluted tubule epithelial cells and microglial cells from the Droplet and FACS batches, respectively–without overcorrecting them by merging with cell types from the opposite batch. scDecorr also forms a well-defined cluster for pancreatic B cells; while a small subset of these cells is disconnected from the main cluster, Leiden clustering correctly assigns them to the same population (region highlighted in purple).

scDecorr forms well-defined clusters for kidney proximal convoluted tubule epithelial cells and enterocytes of the epithelium of the large intestine, although Leiden does not identify them as distinct clusters. Similarly, scDecorr forms visually distinct clusters for endothelial cells of the coronary artery and endothelial cells; however, due to partial mixing near cluster boundaries, Leiden detects these two cell types as a single cluster. scDecorr also partially resolves mesenchymal stem cells, fibroblasts of cardiac tissue, and bladder cells, with some boundary overlap resulting in Leiden grouping these three populations together. Notably, in these cases, most competing methods fail to recover any meaningful separation,with the corresponding cell states being fully intermixed and lacking any discernible separation.

Consistent with other methods, scDecorr merges macrophage, myeloid cell, and promonocyte populations. In addition, a common limitation observed across all methods is the failure to fully integrate granulocytopoietic and granulocyte cell types within the Droplet batch, with these populations unable to form well-defined connected clusters

scCobra clearly clusters B cells, pancreatic B cells, kidney proximal convoluted tubule epithelial cells, enterocytes of the epithelium of the large intestine, hepatocytes, and bladder urothelial cells. However, it completely merges endothelial cells of the coronary artery, endothelial cells, and bronchial smooth muscle cells. Moreover, mesenchymal stem cells, fibroblasts of cardiac tissue, and bladder cells are fully intermixed. The qualitative performance of SCALEX closely resembles that of scCobra.

scVI distinctly clusters B cells, pancreatic B cells, kidney proximal convoluted tubule epithelial cells, enterocytes of the epithelium of the large intestine, hepatocytes, and bronchial smooth muscle cells. In addition, scVI successfully separates skeletal muscle satellite cells, bladder urothelial cells, and basal cells, and resolves mesenchymal stem cells, fibroblasts of cardiac tissue, and bladder cells more effectively than SCALEX and scCobra. However, similar to other methods, scVI merges macrophage, myeloid cell, and promonocyte populations. Although scVI distinguishes endothelial cells of the coronary artery from endothelial cells better than SCALEX and scCobra, endothelial cells from the FACS batch are not properly mixed with those from the Droplet batch, resulting in a disconnected cluster (region marked in violet in Figure S14A).

Harmony identifies major cell populations such as B cells, pancreatic B cells, hepatocytes, bronchial smooth muscle cells, skeletal muscle satellite cells, and bladder urothelial cells. As with most methods, Harmony incorrectly merges macrophage, myeloid cell, and promonocyte populations. Unlike other approaches, however, Harmony exhibits mild overcorrection. Kidney proximal convoluted tubule epithelial cells, a Droplet-specific population, are partially mixed with FACS-derived cell types. Furthermore, the microglial cell cluster, a FACS-specific population, is not well separated and becomes merged with a large population of macrophages from the Droplet batch.

Similar to Harmony, LIGER also overcorrects batch effects. In particular, kidney proximal convoluted tubule epithelial cells from the Droplet batch are incorrectly merged with enterocytes of the epithelium of the large intestine from the FACS batch. Additionally, the microglial cell cluster from the FACS batch is merged with a large macrophage population.

trVAE distinctly clusters most cell types and preserves batch-specific populations. However, similar to scVI, trVAE exhibits mild undercorrection, as endothelial cells from the FACS batch are not fully integrated with those from the Droplet batch, resulting in a discontinuous cluster (region marked in violet in Figure S14A).

In summary, across both the Cross-tissue Immune Atlas and Tabula Muris datasets, scDecorr consistently demonstrates strong and reliable data integration performance by prioritizing biological fidelity while achieving competitive batch correction. scDecorr effectively preserves biologically meaningful structure, producing well-defined, connected clusters and maintaining clear separation between closely related cell states. Importantly, it avoids over-merging batch-specific cell types and prevents small or rare populations from being absorbed into larger clusters, a common failure mode of many competing methods.

Quantitatively, scDecorr achieves the highest biological conservation and overcorrection scores across both datasets, indicating robust preservation of cell identity and minimal distortion of biological signal. Although scDecorr does not always attain the strongest batch mixing, its batch correction performance remains competitive with state-of-the-art methods and substantially exceeds that of unintegrated PCA. By deliberately favoring biological conservation over aggressive batch alignment, scDecorr avoids overcorrection and yields integrated embeddings that are both interpretable and biologically coherent.

### scDecorr corrects batches without overcorrection

The goal of batch correction is to minimize technical batch effects such that true biological structure can be accurately recovered. However, aggressive batch correction can lead to overcorrection, resulting in the unintended mixing of biologically distinct cell states and loss of meaningful biological signal–a persistent challenge across many data integration methods. In this section, we demonstrate that scDecorr effectively corrects batch effects without overcorrection. We evaluate its performance on two datasets, namely the Human Lung and Human Pancreas datasets, which present particularly challenging integration scenarios due to minimal overlap between batches and the presence of multiple batch-specific cell types. We show that scDecorr successfully mixes shared cell populations while preserving batch-specific cell types, thereby avoiding overcompensation during batch correction.

As shown in Fig. 4B, scDecorr achieves the strongest overall overcorrection performance across all evaluated datasets. The next best-performing methods are scVI, Harmony, and scCobra. Notably, PCA also ranks highly in terms of overcorrection score; however, this behavior is expected, as PCA largely avoids batch mixing altogether, as reflected by its low batch entropy scores in Fig. 4E. Consistent with this observation, PCA also exhibits consistently lower ARI scores compared to other methods (Fig. 4D), indicating poor recovery of biological structure.

While scDecorr does not achieve the highest batch entropy scores, its performance remains competitive with other state-of-the-art methods (Fig. 4E). In contrast, methods such as LIGER, trVAE, and Scanorama achieve high batch entropy scores but suffer from substantially lower overcorrection scores, reflecting excessive batch mixing. These methods also exhibit comparatively lower ARI scores, indicating compromised biological preservation. By comparison, scDecorr consistently attains higher ARI scores across datasets, underscoring its ability to correct technical batch effects while minimizing overcorrection and preserving biologically relevant structure.

We next discuss these results in detail for the Human Immune and Human Lung datasets.

**Human Immune:** The Human Immune dataset^6^, which includes 33, 506 cells from human bone marrow, presents a challenging data integration task due to its 10 batches and 16 distinct cell types. The dataset is derived from 5 experimental studies–10X, Freytag, Oetjen, Sun, and Villani. The Oetjen study comprises 3 batches, the Sun study includes 4 batches, while the remaining studies contribute 1 batch each, resulting in a total of 10 batches. Most cell types are shared across studies, with the exception of the Oetjen study, which contains 4 batch-specific cell types: CD10+ B cells, Monocyte progenitors, Erythroid progenitors, and Erythrocytes.

From Fig. 3D and Table S5.1, scDecorr achieves the best biological conservation, data integration, and overcorrection performance among all benchmarked methods. In particular, scDecorr exhibits a 7–$8\%$ improvement in $S_{data\_integration}$ (0.82) over the next best-performing methods, Harmony and scVI, which achieve scores of 0.77 and 0.76, respectively (Table S5.1). scDecorr also achieves a $22\%$ higher $S_{bio}$ score (0.95) compared to SCALEX and Harmony, both of which attain scores of 0.78. With respect to overcorrection, scDecorr performs best, achieving a $4\%$ higher $S_{oc}$ score (0.98) than the next best method, scVI, which achieves a score of 0.94 (Table S5.1). While the overall batch correction score of scDecorr (0.50) is comparatively lower, it remains competitive with SCALEX (0.52) and scCobra (0.59). Notably, scDecorr achieves a batch entropy mixing score of 0.34, comparable to SCALEX (0.32), scCobra (0.36), and Scanorama (0.32), and substantially higher than unintegrated PCA (0.14) (Fig. 4E, Table S4.1).

UMAP visualizations in Fig. 4A show that scDecorr robustly clusters major cell populations such as CD14+ Monocytes, CD4+ T cells, and CD20+ B cells, integrating these populations across all studies into well-separated and coherent clusters. scDecorr also clearly distinguishes NK and NKT cells. Batch-specific cell types from the Oetjen study–including CD10+ B cells, Monocyte progenitors, Erythroid progenitors, and Erythrocytes–are preserved without overcorrection and form distinct clusters. A small subset of Erythrocytes appears disconnected from the main cluster; however, Leiden clustering correctly assigns these cells to the same population. In addition, small cell types such as CD10+ B cells, Monocyte progenitors, and Plasmacytoid dendritic cells are preserved and do not merge with larger populations.

scCobra clusters major cell types such as CD14+ Monocytes, CD4+ T cells, and CD20+ B cells; however, a subset of CD14+ Monocytes from the 10X batch remains disconnected from the main cluster, indicating undercorrection (Fig. 4A, region highlighted in violet). A large fraction of Erythrocytes also remains separated from the primary Erythrocyte cluster, resulting in incorrect clustering (Fig. 4A, region highlighted in violet). Moreover, some Erythrocytes become mildly overcorrected and mix with CD4+ T cells from other batches, despite being a batch-specific population of the Oetjen study. While CD10+ B cells are preserved, Plasmacytoid dendritic cells from the Villani study are undercorrected and remain disconnected from the main cluster, leading to incorrect clustering (Fig. 4A, region highlighted in violet).

SCALEX preserves major cell populations such as CD14+ Monocytes, CD4+ T cells, and CD20+ B cells; however, CD8+ T cells from the Oetjen study are not properly integrated with CD8+ T cells from other studies. Similar to scCobra, SCALEX exhibits undercorrection of Plasmacytoid dendritic cells from the Villani study, which remain disconnected from the main cluster. In addition, SCALEX fails to cluster Erythrocytes correctly, with these cells scattered throughout the embedding and a large fraction overcorrected and mixed with CD4+ T cells from other batches. CD10+ B cells are relatively well preserved.

scVI preserves most major cell types but fails to integrate CD14+ Monocytes fully, resulting in a subset of cells separated from the main cluster. Erythrocytes are also poorly integrated, forming discontinuous and scattered clusters. In contrast, scVI preserves CD10+ B cells and Plasmacytoid dendritic cells effectively.

Harmony fails to properly integrate CD14+ Monocytes, producing scattered and suboptimal clusters. It also exhibits substantial overcorrection by mixing batch-specific Oetjen populations, including Erythrocytes and CD10+ B cells, with other batches. Harmony further mixes some Plasmacytoid dendritic cells from the Villani study with CD20+ B cells and fails to distinguish NK and NKT populations.

LIGER incorrectly mixes several cell types, including CD14+ Monocytes with CD16+ Monocytes and monocyte-derived dendritic cells. It also overcorrects CD10+ B cells by merging them with CD20+ B cells. While LIGER preserves the Erythrocyte cluster, it fails to integrate Plasmacytoid dendritic cells properly, resulting in uneven and scattered clusters. In addition, LIGER largely merges NK and NKT populations.

trVAE and Scanorama extensively mix most cell types, with the exception of CD14+ Monocytes and CD20+ B cells, which remain reasonably well preserved (Figure S8A).

**Human Lung**

The Human Lung dataset^6^ consists of 32, 472 cells obtained from three distinct studies: 10x (Transplant), 10x (Biopsy), and Drop-seq (Transplant). Each study includes cells obtained from multiple donors. Specifically, the 10x (Transplant) study contains 12, 725 single cells sequenced from 6 donors identified as $1, 2, \ldots , 6$. The 10x (Biopsy) study includes 10, 046 cells sequenced from 6 donors identified as $A1, A2, \ldots , A6$. The Drop-seq (Transplant) study consists of 9, 701 cells originating from 4 donors identified as $B1, B2, \ldots , B4$. These 16 unique donor identifiers define the batches in this dataset, resulting in a total of 16 batches.

The cell-type composition of the 10x (Biopsy) study (donors *A*1–*A*6) differs substantially from the other studies. This batch is dominated by Ciliated, Basal-1, Basal-2, and Secretory cell types, which appear only in minor proportions or are entirely absent in the remaining batches. We use scDecorr to integrate the Human Lung dataset across all 16 donors.

scDecorr achieves the highest biological conservation score ($S_{bio}$) among all benchmarked methods, with approximately 20–$30\%$ higher mean $S_{bio}$ (0.99) compared to the next best-performing methods, scVI (0.83) and scCobra (0.77) (Table S5.1). With mean ARI and cell-type ASW scores of 0.64 and 0.63, respectively, scDecorr substantially outperforms other methods (Table S4.1, Figure S4.1A,E). By comparison, scVI and scCobra achieve mean ARI scores of 0.57 and 0.55, and mean cell-type ASW scores of 0.55 and 0.54, respectively.

While scDecorr exhibits comparatively lower batch correction strength, its performance remains competitive with other methods. In particular, scDecorr attains a mean batch entropy mixing score of 0.26, comparable to scCobra (0.28), SCALEX (0.27), scVI (0.27), and Harmony (0.28), and substantially higher than unintegrated PCA (0.14) (Table S4.1, Figure S4.1D). scDecorr also achieves a graph connectivity score of 0.98, comparable to scVI (0.98), scCobra (0.96), SCALEX (0.95), and Harmony (0.95), and higher than PCA (0.82) (Table S4.1, Fig. 4.1F). The comparatively lower batch entropy scores of scDecorr reflect its deliberate strategy to minimize overcorrection, which is further supported by its overcorrection performance. scDecorr achieves the highest overcorrection score, exceeding the next best method, scVI, by $6\%$ (0.85 versus 0.80) (Table S4.1, Figure S4.1G). scCobra, SCALEX, and Harmony obtain overcorrection scores of 0.74, 0.74, and 0.77, respectively.

UMAP visualizations in Fig. 4F show that, in scDecorr embeddings, cells from the 10x (Biopsy) experiment exhibit minimal overlap with cells from other experiments. This behavior is expected and desirable given the limited overlap in cell-type composition across these batches. In particular, scDecorr does not overcorrect the batch-specific Basal-1 and Basal-2 cell types from the 10x (Biopsy) study and clusters them distinctly. At the same time, scDecorr successfully integrates major shared cell types, such as Ciliated cells, across batches, resulting in coherent and well-separated clusters. However, scDecorr fails to fully integrate a subset of Secretory cells from the Drop-seq (Transplant) batch with those from the 10x (Biopsy) batch. Small cell populations, including Fibroblast, Lymphatic, and Endothelium cells, are well preserved and form distinct clusters.

scCobra also preserves Basal-1 and Basal-2 cells from the 10x (Biopsy) batch without overcorrection. However, it fails to properly integrate Ciliated cells, with Ciliated cells from the Drop-seq (Transplant) and 10x (Transplant) batches remaining disconnected from those in the 10x (Biopsy) batch. This undercorrection results in fragmented and discontinuous clusters. In addition, scCobra incorrectly mixes Secretory cells with B cells and Endothelium cells with Lymphatic cells.

SCALEX similarly avoids overcorrection of Basal-1 and Basal-2 cells and clusters them well. However, like scCobra, it fails to integrate Ciliated cells across batches and incorrectly merges Endothelium and Lymphatic cell populations.

scVI correctly clusters Basal-1, Basal-2, Secretory, and Ciliated cells, and forms well-separated clusters for Fibroblast, Lymphatic, and Endothelium cell types.

Harmony exhibits overcorrection, merging Type 2 cells from the 10x (Transplant) batch with Basal-1, Basal-2, and Secretory cells from the 10x (Biopsy) batch. Secretory cells are not properly integrated. Nevertheless, Harmony preserves Ciliated, Fibroblast, Lymphatic, and Endothelium cell types.

Similar to Harmony, LIGER overcorrects the 10x (Biopsy) batch, merging Basal-1, Basal-2, and Secretory cells with Type 2 cells from the 10x (Transplant) batch. LIGER also fails to integrate Ciliated cells effectively, resulting in fragmented clusters, but preserves Fibroblast, Lymphatic, and Endothelium populations.

Overall, scDecorr achieves a balanced integration on the Human Lung dataset. It avoids overcorrecting the 10x (Biopsy) batch, which has limited overlap with other batches, thereby preserving batch-specific cell types such as Basal-1 and Basal-2. Unlike SCALEX and scCobra, scDecorr also avoids undercorrection of shared cell types such as Ciliated cells, even when these populations are disproportionately represented in a single batch. In addition, scDecorr consistently preserves small cell populations, including Fibroblast, Lymphatic, and Endothelium cells, resulting in biologically coherent and interpretable embeddings.

In summary, scDecorr consistently strikes an effective balance between batch correction and biological preservation. Rather than enforcing aggressive batch alignment in settings with limited overlap, scDecorr selectively integrates shared cell populations while preserving batch-specific and rare cell types. This strategy minimizes inappropriate cell-type mixing and reduces the risk of overcorrection, leading to integrated embeddings that retain biologically meaningful structure. By combining competitive batch correction with consistently high biological conservation and overcorrection performance, scDecorr provides a robust and reliable framework for scRNA-seq data integration that prioritizes biological fidelity.

### scDecorr outperforms other methods in label transfer

**Fig. 5:**
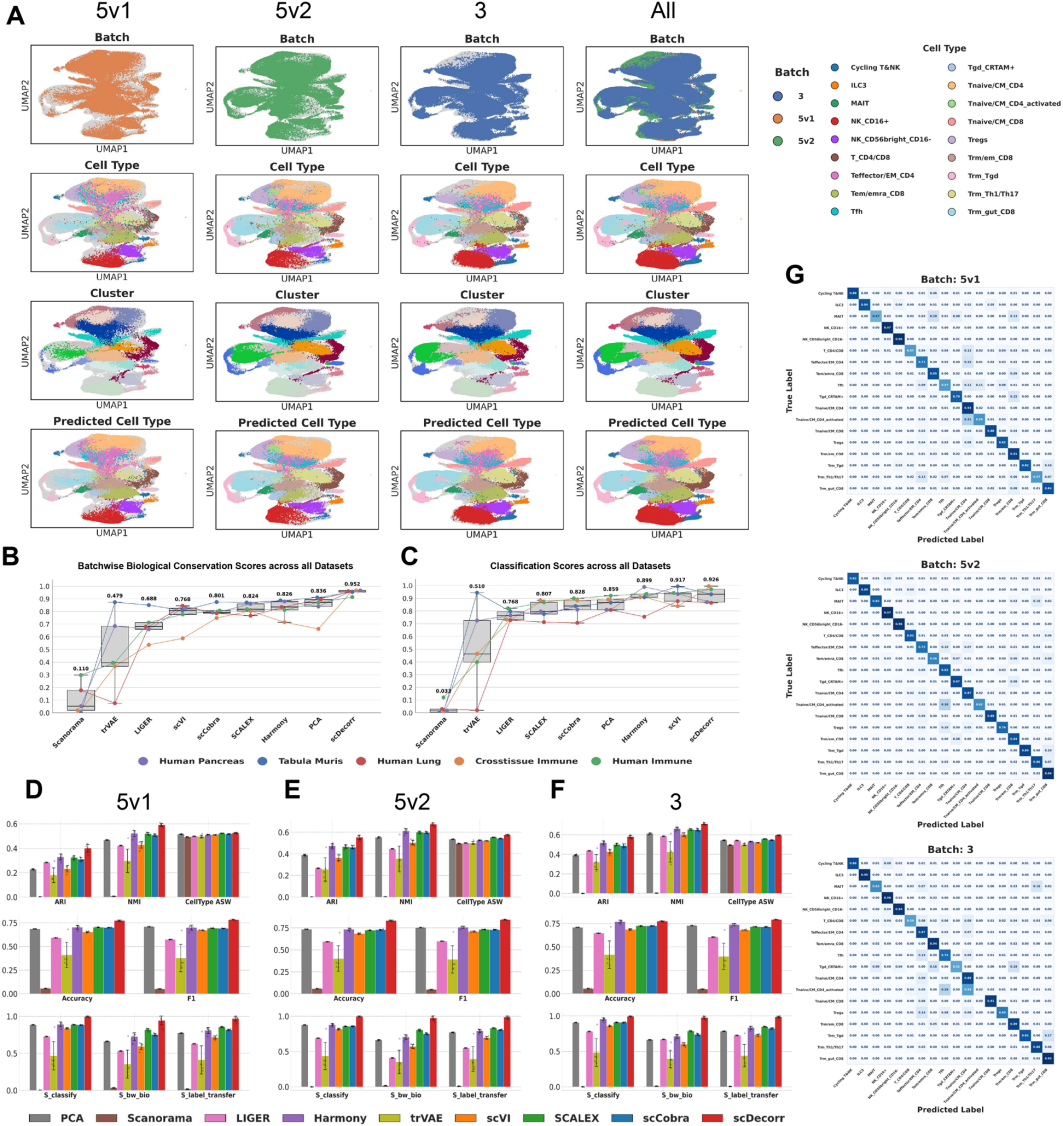
Label transfer performance of benchmark methods: A) Batch-wise UMAP plots of scDecorr on cross-tissue immune dataset. B) Boxplots denoting batch-wise biological conservation performance ($S_{bw\_bio}$) of methods on all benchmarking datasets, C) Boxplots denoting batch-wise classification performance ($S_{classify}$) of methods on all benchmarking datasets, D, E, F) Barplots indicating label transfer scores of all benchmark methods on 5v1, 5v2 and 3 batches of crosstissue immune dataset respectively, G) Confusion matrices indicate the agreement between ground-truth and predicted cell-type labels (TPR) obtained by scDecorr after batch-wise label transfer on the cross-tissue immune atlas (values normalized across rows).

We next evaluate the label transfer performance of scDecorr. As shown in Fig. 5B and Fig. 5C, scDecorr achieves the strongest overall performance across datasets in terms of both classification accuracy and batch-wise biological conservation. For classification accuracy, scVI and Harmony are the next best-performing methods, while PCA and Harmony perform relatively well in terms of batch-wise biological conservation. Notably, scDecorr outperforms all benchmark methods by a substantial margin in batch-wise biological conservation across all datasets.

With respect to classification performance, scDecorr achieves the best results on the Cross-tissue Immune and Human Immune datasets and remains competitive with other methods on the remaining datasets. Overall, the performance of most benchmark methods is stable across datasets, with the notable exception of trVAE, which performs poorly on the Cross-tissue Immune, Human Immune, and Human Lung datasets.

Consistent with these observations, Figure S4.2B shows that scDecorr attains the highest mean F1 scores (averaged across batches) on the Human Lung, Cross-tissue Immune, and Human Immune datasets. On the Human Pancreas and Tabula Muris datasets, scDecorr does not achieve the top F1 score but remains competitive with other leading methods. Furthermore, Figure S4.2C demonstrates that scDecorr consistently outperforms all benchmark methods in terms of mean batch-wise ARI across all datasets, highlighting its superior ability to transfer labels while preserving biologically meaningful structure across batches.

We next examine label transfer performance in greater detail on selected benchmark datasets. As noted earlier, label transfer is performed across distinct experimental sources rather than individual donor batches.

**Crosstissue Immune:** We perform label transfer across the 5v1, 5v2, and 3 chemistries. From Fig. 5D, E, and F, scDecorr substantially outperforms all benchmark methods in both classification accuracy and batch-wise biological conservation across all three chemistries. scDecorr achieves a mean $S_{label\_transfer}$ of 0.98 across chemistries, markedly exceeding the next best-performing methods, SCALEX (0.84) and scCobra (0.81) (Table S7.2). Classification performance is likewise strongest for scDecorr, with a perfect mean $S_{classify}$ score of 1.0, followed by Harmony (0.91) and PCA (0.89) (Table S7.2). Batch-wise biological conservation ($S_{bw\_bio}$) is also highest for scDecorr, with a mean score of 0.97, compared to 0.80 for SCALEX and 0.75 for scCobra (Table S7.2). In addition, scDecorr’s performance is stable across multiple random seed runs.

UMAP visualizations of predicted cell types produced by scDecorr across all chemistries are shown in Fig. 5A, while corresponding predictions for other benchmark methods are shown in Fig. 3A. Figure 5F presents classification confusion matrices for scDecorr, with values corresponding to true positive rate (TPR; recall). When performing label transfer on the 5*v*1 batch, scDecorr accurately classifies several major cell types, including Cyclic T&NK, NK_$CD16+$, NK_CD56bright_CD16-, and Tnaive/CM_CD4 cells, achieving TPRs of 0.88, 0.97, 0.96, and 0.93, respectively (Fig. 5F). In contrast, MAIT cells are frequently misclassified as Tem/emra_CD8 and Trm/em_CD8, resulting in a lower TPR of 0.47.

During label transfer on the 5*v*2 batch, scDecorr correctly classifies most cell types, with the primary exception of Tnaive/CM_CD4_activated cells, a substantial fraction of which are misclassified as Tfh cells. When using the 3 chemistry as the query batch, scDecorr accurately classifies Cyclic T&NK, ILC3, NK_CD16+, NK_CD56bright_CD16−, Tnaive/CM_CD8, and Trm_gut_CD8 populations. However, classification performance for Tnaive/CM_CD4_activated cells remains limited, with many cells misclassified as Tnaive/CM_CD4.

Finally, we evaluate inter-organ label transfer using the integrated representations generated by scDecorr. In this analysis, cells from one organ are treated as queries while cells from the remaining organs serve as references. UMAP visualizations of organ-wise annotations produced by scDecorr are shown in Figure S16, and corresponding quantitative benchmarks are presented in Figure S17. Across all organs, scDecorr consistently outperforms competing methods in both clustering and classification metrics. These results further demonstrate the robustness and effectiveness of scDecorr for accurate label transfer across diverse tissue sources, underscoring its utility for large-scale integrative analyses.

**Human Immune:** For this dataset, we perform label transfer across the 5 experimental studies, namely Oetjen, Freytag, 10X, Sun, and Villani. Figure S9C summarizes the classification and batch-wise biological conservation scores for scDecorr and other benchmark methods. scDecorr achieves the best mean label transfer performance across all studies among all methods (Table S7.2). Specifically, scDecorr attains a mean $S_{label\_transfer}$ of 0.94, outperforming the next best methods, PCA (0.90) and Harmony (0.87). In terms of classification accuracy, scDecorr again performs best, achieving a mean $S_{classify}$ of 0.97, compared to scVI (0.94) and Harmony (0.93) (Table S7.2).

Consistent with these results, scDecorr achieves the highest mean F1 score (0.81) and balanced accuracy (0.84) among all benchmark methods, with the next best performance obtained by scVI (mean F1 of 0.79 and balanced accuracy of 0.82; Figure S4.2B,D). scDecorr also outperforms all other methods in terms of batch-wise biological conservation. Batch-wise predicted cell types produced by scDecorr and the corresponding confusion matrices are shown in Figure S9A and Figure S9B, respectively.

From Figure S9B, scDecorr accurately classifies most cell types during label transfer to the 10X study, including CD14+ Monocytes, CD20+ B cells, CD4+ T cells, Megakaryocyte progenitors, and Plasmacytoid dendritic cells, achieving true positive rates (TPR) in the range of 0.85–1.0. An exception is Plasma cells, many of which are misclassified as CD20+ B cells. During label transfer to the Freytag study, most major cell types–such as CD14+ Monocytes, CD16+ Monocytes, CD20+ B cells, and CD4+ T cells–are classified accurately, although a subset of CD8+ T cells is misclassified as CD4+ T cells.

For label transfer to the Oetjen study, which has the least overlap with other batches, scDecorr correctly classifies major shared populations, including CD14+ Monocytes, CD16+ Monocytes, CD20+ B cells, and CD4+ T cells. However, CD8+ T cells, the majority of which originate from the Oetjen study, are frequently misclassified as CD4+ T cells. Notably, hematopoietic stem and progenitor cells (HSPCs), more than $90\%$ of which originate from the Oetjen study, are still classified effectively by scDecorr, achieving a TPR of 0.90. The precision for HSPC classification is lower, however, as a substantial number of Megakaryocyte progenitors are misclassified as HSPCs.

The Villani study contains only 4 cell types shared with other batches–CD14+ Monocytes, CD16+ Monocytes, monocyte-derived dendritic cells, and Plasmacytoid dendritic cells. Among these, CD14+ Monocytes, monocyte-derived dendritic cells, and Plasmacytoid dendritic cells are classified accurately by scDecorr, whereas a large fraction of CD16+ Monocytes is misclassified as CD14+ Monocytes. Finally, during label transfer to the Sun study, scDecorr accurately classifies all cell types.

Overall, these results demonstrate that scDecorr consistently achieves strong label transfer performance across diverse experimental sources, including challenging settings with limited batch overlap, while maintaining high classification accuracy and batch-wise biological conservation.

**Human Lung:** We perform label transfer across the 3 experimental studies–10x Biopsy, 10x Transplant, and Drop-seq Transplant–on the Human Lung dataset. Figure S11A summarizes the label transfer performance of all benchmark methods. Overall, scDecorr achieves the strongest mean label transfer performance across batches among all methods. Specifically, scDecorr attains a mean $S_{label\_transfer}$ score of 0.91 across all batches, outperforming other benchmark methods, with the next best-performing method being scVI, which achieves a mean score of 0.86 (Table S7.2).

In terms of classification performance ($S_{classify}$), scDecorr achieves a mean score of 0.86, which is competitive with scVI (0.87) and higher than that of the remaining methods (Table S7.2). With respect to batch-wise biological conservation, scDecorr outperforms all benchmark methods by a substantial margin. Consistent with this observation, Figure S4.2B and Table S6.1 show that scDecorr achieves the highest mean F1 score across all batches, while its mean accuracy remains competitive with other leading methods.

Batch-wise UMAP visualizations and corresponding confusion matrices for scDecorr are shown in Figure S11B and Figure S11C, respectively, while corresponding predictions for other benchmark methods are shown in Figure S10A. During label transfer to the Drop-seq Transplant study, scDecorr accurately classifies major cell types, including Macrophage, Type 2, and T/NK cells, achieving true positive rates (TPR) of 0.92, 0.89, and 0.93, respectively (Figure S11C). Rare cell types such as Endothelium, Fibroblast, Lymphatic, and Mast cells are also classified effectively, with TPRs of 0.92, 0.82, 0.84, and 0.84, respectively. In contrast, scDecorr performs poorly on Secretory cells in this setting, with the majority of these cells misclassified as Macrophage or Type 2 cells.

When performing label transfer on the 10x Biopsy study, which exhibits the least overlap with other batches, most shared cell types–including Ciliated, Endothelium, and Fibroblast cells–are classified accurately, with TPRs ranging from 0.95 to 1.0. However, cell types that originate predominantly from the 10x Biopsy batch, such as Basal1 and Basal2, are not classified accurately. In addition, Secretory cells, the majority of which come from the 10x Biopsy study, are frequently misclassified as Ciliated cells.

For label transfer on the 10x Transplant batch, scDecorr accurately classifies Ciliated, T/NK, Type 2, Endothelium, Fibroblast, and Lymphatic cell types. However, Neutrophil populations–specifically Neutrophil_CD14_high and Neutrophil_IL1R2–which predominantly originate from the 10x Transplant study, are not classified accurately.

Overall, these results indicate that scDecorr achieves strong and consistent label transfer performance on the Human Lung dataset, particularly for shared and rare cell types, while challenges remain for batch-specific populations with limited representation in reference datasets.

**Tabula Muris:** We perform label transfer across the Droplet and FACS technologies in the Tabula Muris dataset. Figure S15C summarizes the label transfer performance of all benchmark methods across these 2 batches. scDecorr outperforms all other methods in terms of overall (mean) label transfer performance ($S_{label\_transfer}$) across Droplet and FACS. Specifically, scDecorr achieves a mean $S_{label\_transfer}$ of 0.94, exceeding the performance of the next best methods, trVAE (0.91) and scVI (0.90) (Table S7.2).

With respect to classification performance alone, scDecorr does not achieve the highest score but remains competitive with other methods. scVI performs best in terms of classification, achieving an $S_{classify}$ of 0.99, followed by trVAE (0.94) and scDecorr (0.93) (Table S7.2). In contrast, scDecorr clearly outperforms all benchmark methods in terms of batch-wise biological conservation. Consistent with this observation, Figure S4.2B,D and Table S6.1 show that the mean F1 score and mean accuracy of scDecorr across all batches are competitive with those of other leading methods.

Batch-wise UMAP visualizations and confusion matrices for scDecorr are shown in Figure S15A and Figure S15B, respectively, while the corresponding predictions for other benchmark methods are shown in Figure S14A. During label transfer to the Droplet batch, several cell types–including B cells, basal cells, bladder urothelial cells, hepatocytes, pancreatic B cells, and skeletal muscle satellite cells–are classified accurately, with true positive rates (TPR) exceeding 0.90 (Figure S15B). In contrast, T cells, classical monocytes, granulocytopoietic cells, and macrophages are not classified accurately in this setting.

For label transfer to the FACS batch, scDecorr accurately classifies B cells, basal cells, bladder urothelial cells, basal cells of the epidermis, granulocytopoietic cells, mesenchymal stem cells, hepatocytes, and bronchial smooth muscle cells. However, granulocytes, DN4 thymocytes, mesenchymal stem cells of adipose tissue, and monocytes are not classified accurately.

Finally, we evaluate inter-organ label transfer on the Tabula Muris dataset. In this setting, cells from one organ are treated as queries, while cells from the remaining organs serve as references. UMAP visualizations (Figure S18) and corresponding benchmark results (Figure S19) demonstrate that scDecorr consistently achieves high annotation performance across all organs, outperforming or matching other methods in both clustering and classification metrics. Notably, scDecorr preserves organ-specific cellular identities while enabling accurate label transfer across organs.

**Human Pancreas.** We perform label transfer across 6 experimental batches–*CEL-Seq*, *CEL-Seq*2, *Fluidigm C1*, *inDrop*, *Smart-Seq*, and *Smart-Seq*2–representing diverse sequencing protocols and coverage profiles in the Human Pancreas dataset. Figure S13A summarizes the label transfer performance of all benchmark methods on this dataset. Across all batches, scDecorr achieves strong and stable label transfer performance, remaining competitive with the best-performing benchmark methods in terms of classification accuracy while consistently excelling in batch-wise biological conservation.

From Table S7.2, Harmony achieves the highest overall label transfer performance with an $S_{label_transfer}$ score of 0.93, followed by scDecorr (0.92) and scVI (0.88). With respect to classification performance, Harmony again performs best, attaining an $S_{classify}$ score of 0.98, followed by scVI (0.93) and scDecorr (0.87). Consistent with these results, Figure S4.2 and Table S6.1 show that the mean F1 score and mean accuracy of scDecorr across all batches are competitive with those of other leading methods. Batch-wise UMAP visualizations and confusion matrices for scDecorr are shown in Figure S13B and Figure S13C, respectively, while predicted cell types for other benchmark methods are shown in Figure S12A.

From the batch-wise classification results, scDecorr accurately transfers labels for major pancreatic cell populations across all technologies. In particular, endocrine cell types such as *alpha*, *beta*, *delta*, and *gamma* cells are classified with high fidelity across all batches, with F1 scores typically exceeding 0.95 in CEL-Seq2, Fluidigm C1, Smart-Seq, and Smart-Seq2, and remaining robust in the larger and noisier inDrop batch. Similarly, abundant exocrine and structural cell types, including *acinar* and *ductal* cells, are consistently identified with high recall across most batches, demonstrating effective alignment of shared biological structure despite strong protocol-induced batch effects.

Performance degradation is primarily observed for rare cell populations with extremely low abundance or strong batch specificity. Across multiple batches, cell types such as *epsilon*, *quiescent stellate*, *Schwann*, *mast*, and *macrophage* cells exhibit reduced recall and F1 scores, largely due to their scarcity and limited representation in reference batches. For example, *epsilon* cells are frequently misclassified in CEL-Seq, CEL-Seq2, Fluidigm C1, and inDrop, while *quiescent stellate* cells show poor recall in inDrop and Fluidigm C1. These trends are consistent with observations across other datasets and highlight the inherent difficulty of transferring labels for rare populations under strong batch imbalance.

Despite these challenges, scDecorr maintains high overall accuracy across all batches, ranging approximately from 0.92 to 0.99, and achieves robust F1 scores, particularly in larger and more complex batches such as inDrop and CEL-Seq2. Importantly, the strong batch-wise biological conservation achieved by scDecorr indicates that the learned integrated representations preserve meaningful cell-type structure within each batch while enabling accurate cross-batch annotation. Collectively, these results demonstrate that scDecorr provides reliable and biologically coherent label transfer across heterogeneous pancreatic scRNA-seq datasets, effectively balancing classification accuracy with batch-wise structural preservation.

Across all evaluated datasets, scDecorr demonstrates consistently strong label transfer performance by combining competitive classification accuracy with superior batch-wise biological conservation. Its robustness across diverse sequencing technologies, tissue types, and batch structures highlights scDecorr as a reliable framework for large-scale scRNA-seq data integration and downstream cell-type annotation.

On the Cross-tissue Immune dataset, scDecorr clearly outperforms all benchmark methods in both classification accuracy and batch-wise biological conservation across multiple sequencing chemistries. For the Human Immune dataset, scDecorr achieves the best overall label transfer performance, surpassing other methods in classification accuracy, F1 score, and biological preservation across studies. In the Human Lung dataset, scDecorr attains the highest overall label transfer and batch-wise conservation scores while remaining competitive in classification despite strong batch heterogeneity. On the Tabula Muris dataset, scDecorr delivers the strongest overall label transfer performance across Droplet and FACS technologies and achieves the highest batch-wise biological conservation with comparable classification accuracy. In the Human Pancreas dataset, scDecorr remains competitive with leading methods in classification while consistently providing stronger batch-wise biological conservation across diverse sequencing protocols.

Collectively, these results demonstrate that scDecorr effectively balances accurate cross-batch cell-type annotation with preservation of within-batch biological structure, enabling robust and biologically coherent label transfer across heterogeneous scRNA-seq datasets.

## Discussion

To facilitate the learning of biologically meaningful latent representations from unannotated single-cell data, scDecorr adopts a self-supervised learning strategy that maximizes alignment between joint embeddings of distorted gene expression profiles while simultaneously decorrelating their components. This objective serves two complementary purposes. First, by enforcing similarity between embeddings derived from independently distorted views of the same cell, scDecorr learns representations that are invariant to stochastic perturbations of gene expression, thereby improving robustness to technical noise. Second, by decorrelating the components of the embedding vectors, scDecorr encourages the latent space to capture diverse and non-redundant biological signals, enabling effective representation learning without relying on negative samples. Together, these design choices allow scDecorr to learn expressive and stable embeddings in a fully self-supervised manner.

In the context of integrating single-cell data across multiple domains–including donors, studies, sequencing chemistries, platforms, and species–scDecorr leverages domain adaptation to learn domain-invariant representations. Rather than explicitly correcting batch effects, scDecorr independently samples cells from each domain and employs domain-specific batch normalization layers during training. This strategy enables the model to learn a shared latent space that is simultaneously optimized across domains, without enforcing hard alignment constraints. As a result, scDecorr implicitly mixes batches while preserving domain-specific biological structure. This contrasts with many existing integration methods that rely on explicit batch correction, which can often lead to overcorrection and loss of biologically meaningful variation.

The empirical results across diverse datasets demonstrate that this implicit integration strategy yields tangible benefits. scDecorr consistently achieves strong biological conservation, forming well-defined and connected clusters for shared cell types while preserving batch-specific and rare populations. In challenging integration scenarios characterized by limited batch overlap or strong asymmetry–such as the Cross-tissue Immune, Human Lung, and Human Immune datasets–scDecorr avoids aggressive mixing of unrelated cell states and minimizes overcorrection. This behavior is reflected in its consistently high overcorrection scores and competitive batch-mixing performance, indicating a favorable balance between technical correction and biological fidelity.

Beyond data integration, the learned representations produced by scDecorr exhibit strong robustness in downstream label transfer tasks. Across multiple datasets and experimental settings, scDecorr enables accurate cross-batch, cross-study, and cross-organ label transfer, often outperforming or matching state-of-the-art methods in classification accuracy while consistently achieving superior batch-wise biological conservation. Notably, scDecorr performs particularly well in settings involving heterogeneous sequencing protocols and complex batch structures, highlighting the generality of its learned latent space. These results suggest that scDecorr’s representations capture biologically meaningful structure that generalizes beyond the specific conditions observed during training.

Despite these strengths, scDecorr’s reliance on implicit batch alignment introduces certain limitations. In scenarios with extreme batch imbalance or near-complete non-overlap in cell-type composition, the absence of explicit alignment objectives can lead to reduced performance, particularly for batch-specific or poorly represented populations. While scDecorr mitigates overcorrection in such settings, some degree of underintegration may occur when shared biological signal is minimal. Addressing these challenges represents an important direction for future work. Potential extensions include incorporating stronger domain adaptation mechanisms, such as adversarial training or gradient reversal layers, to encourage more explicit alignment when appropriate. Another promising direction is to introduce batch identifiers as trainable embeddings, allowing the model to condition latent representations on batch-specific context while retaining the benefits of self-supervised learning. Moreover, in its current formulation, the intended use case of scDecorr is offline integration of a fixed set of batches (static datasets). When new domains are introduced, additional training or fine-tuning is generally required to obtain appropriate domain-specific normalization statistics. Extending the framework toward continual or online integration is an important direction for future work.

In summary, scDecorr introduces a principled alternative to explicit batch correction by combining self-supervised representation learning with implicit domain adaptation. Its ability to balance batch mixing and biological preservation across a wide range of integration and label transfer tasks makes it a robust and flexible framework for large-scale scRNA-seq analysis. By prioritizing biological fidelity and avoiding aggressive alignment, scDecorr provides a reliable foundation for downstream analyses that depend on accurate and interpretable cellular representations.

## Supplementary Information

Supplementary Information 1.

Supplementary Information 2.

Supplementary Information 3.

Supplementary Information 4.

Supplementary Information 5.

Supplementary Information 6.

Supplementary Information 7.

Supplementary Information 8.

Supplementary Information 9.

## Data Availability

The processed human lung dataset was obtained from Luecken et al.^6^, who compiled and processed the data using publicly available Drop-seq batch (GSE130148) (Gene Expression Omnibus GEO) and 10x data from Vieira Braga et al.^50^. The processed version of human lung data is available at https://figshare.com/ndownloader/files/24539942. Human Immune dataset was obtained from Luecken et al.^6^, who processed and curated the data using https://support.10xgenomics.com/single-cell-gene-expression/datasets/3.0.0/pbmc_10k_v3, GSE115189, GSE128066, and GSE94820 (GEO). The processed human immune data is available at https://figshare.com/ndownloader/files/25717328. The human pancreas data is also obtained from Luecken et al.^6^, who compiled and processed the data from GSE81076, GSE85241, GSE86469, GSE84133, GSE81608 (GEO), and E-MTAB-5061 (Array Express). The processed data is available at https://figshare.com/ndownloader/files/24539828. For the Tabula Muris dataset^3^, single-cell data from both FACS and Droplet batches can be accessed at https://figshare.com/projects/Tabula_Muris_Transcriptomic_characterization_of_20_organs_and_tissues_from_Mus_musculus_at_single_cell_resolution/27733. The Cross-Tissue Immune Atlas^42^ is publicly available at: https://www.tissueimmunecellatlas.org/. We used the T-cells population of this atlas which is available at: https://cellgeni.cog.sanger.ac.uk/pan-immune/t-cells.h5ad.

## References

[1] Lawson, D. A., Kessenbrock, K., Davis, R. T., Pervolarakis, N. & Werb, Z. Tumour heterogeneity and metastasis at single-cell resolution. *Nat. Cell Biol.* **20**(12), 1349–1360 (2018). 30482943 10.1038/s41556-018-0236-7 PMC6477686

[2] Cao, Z.-J. & Gao, G. Multi-omics single-cell data integration and regulatory inference with graph-linked embedding. *Nat. Biotechnol.* **40**(10), 1458–1466 (2022). 35501393 10.1038/s41587-022-01284-4 PMC9546775

[3] Tabula Muris Consortium & Overall coordination. Single-cell transcriptomics of 20 mouse organs creates a tabula muris. *Nature* **562**(7727), 367–372 (2018). 10.1038/s41586-018-0590-4 PMC6642641 30283141

[4] Yang, X., Baumgart, S. J., Stegmann, C. M. & Hayat, S. Maca: Marker-based automatic cell-type annotation for single-cell expression data. *Bioinformatics* **38**(6), 1756–1760 (2022). 34935911 10.1093/bioinformatics/btab840

[5] Yang, X., Kramann, R., McCord, R. P. & Hayat, S. Masi enables fast model-free standardization and integration of single-cell transcriptomics data. *Commun. Biol.* **6**(1), 465 (2023). 37117305 10.1038/s42003-023-04820-3 PMC10144903

[6] Luecken, M. D. et al. Benchmarking atlas-level data integration in single-cell genomics. *Nat. Methods.* **19**(1), 41–50 (2022). 34949812 10.1038/s41592-021-01336-8 PMC8748196

[7] Tran, H. T. N. et al. A benchmark of batch-effect correction methods for single-cell RNA sequencing data. *Genome Biol.* **21**, 1–32 (2020). 10.1186/s13059-019-1850-9 PMC6964114 31948481

[8] Xu, Y. & Hayat, S. Masiv2 enables standardization and integration of multi-modal single-cell and spatial omics data with one general framework. *bioRxiv* 2023–05 (2023).

[9] Satija, R., Farrell, J. A., Gennert, D., Schier, A. F. & Regev, A. Spatial reconstruction of single-cell gene expression data. *Nat. Biotechnol.* **33**(5), 495–502 (2015). 25867923 10.1038/nbt.3192 PMC4430369

[10] Korsunsky, I. et al. Fast, sensitive and accurate integration of single-cell data with Harmony. *Nat. Methods.* **16**(12), 1289–1296 (2019). 31740819 10.1038/s41592-019-0619-0 PMC6884693

[11] Haghverdi, L., Lun, A. T., Morgan, M. D. & Marioni, J. C. Batch effects in single-cell RNA-sequencing data are corrected by matching mutual nearest neighbors. *Nat. Biotechnol.* **36**(5), 421–427 (2018). 29608177 10.1038/nbt.4091 PMC6152897

[12] Lopez, R., Regier, J., Cole, M. B., Jordan, M. I. & Yosef, N. Deep generative modeling for single-cell transcriptomics. *Nat. Methods.* **15**(12), 1053–1058 (2018). 30504886 10.1038/s41592-018-0229-2 PMC6289068

[13] Chen, Ting, Kornblith, Simon, Norouzi, Mohammad, & Hinton, Geoffrey. A simple framework for contrastivelearning of visual representations. In *International conference on machine learning*, pages 1597–1607. (PMLR, 2020).

[14] Lan, Z. et al. Albert: Alite bert for self-supervised learning of language representations. arXiv preprint arXiv:1909.11942, (2019).

[15] Baevski, A., Zhou, Y., Mohamed, A. & Auli, Michael. wav2vec 2.0: A framework for self-supervised learning of speech representations. *Adv. Neural Inf. Process. Syst.* **33**, 12449–12460 (2020).

[16] Grill, J.-B. et al. Bootstrap your own latent-a new approach to self-supervised learning. *Adv. Neural Inf. Process. Syst.* **33**, 21271–21284 (2020).

[17] Zhao, J., Zhang, Y., He, X. & Xie, P. Covid-ct-dataset: a ct scan dataset about covid-19. (2020).

[18] Ciortan, M. & Defrance, M. Contrastive self-supervised clustering of scRNA-seq data. *BMC Bioinformatics* **22**(1), 280 (2021). 34044773 10.1186/s12859-021-04210-8 PMC8157426

[19] Shen, H. et al. Miscell: An efficient self-supervised learning approach for dissecting single-cell transcriptome. *iScience* **24**(11), 103200 (2021). 34712916 10.1016/j.isci.2021.103200 PMC8529514

[20] Yan, X., Zheng, R. & Li, M. Globe: A contrastive learning-based framework for integrating single-cell transcriptome datasets. *Brief. Bioinform.*10.1093/bib/bbac311 (2022). 35901449 10.1093/bib/bbac311

[21] Yan, X., Zheng, R., Fangxiang, W. & Li, M. Claire: Contrastive learning-based batch correction framework for better balance between batch mixing and preservation of cellular heterogeneity. *Bioinformatics* **39**(3), btad099 (2023). 36821425 10.1093/bioinformatics/btad099 PMC9985174

[22] Yang, X., Das, P. & McCord, R. P. Smile: Mutual information learning for integration of single-cell omics data. *Bioinformatics* **38**(2), 476–486 (2022). 34623402 10.1093/bioinformatics/btab706 PMC10060712

[23] van den Oord, A., Li, Y. & Vinyals, O. Representation learning with contrastive predictive coding. arXiv preprint arXiv:1807.03748, (2018).

[24] Xiaokang, Y., Xinyi, X., Zhang, J. & Li, X. Batch alignment of single-cell transcriptomics data using deep metric learning. *Nat. Commun.* **14**(1), 960 (2023). 36810607 10.1038/s41467-023-36635-5 PMC9944958

[25] Han, W. et al. Self-supervised contrastive learning for integrative single cell RNA-seq data analysis. *Brief. Bioinform.* **23**(5), bbac377 (2022). 36089561 10.1093/bib/bbac377 PMC9487595

[26] He, K., Fan, H., Wu, Y., Xie, S. & Girshick, R. Momentum contrast for unsupervised visual representation learning. In *Proceedings of the IEEE/CVF conference on computer vision and pattern recognition*, pages 9729–9738, (2020).

[27] Thota, M. & Leontidis, G. Contrastive domain adaptation. In *Proceedings of the IEEE/CVF Conference on Computer Vision and Pattern Recognition*, pages 2209–2218, (2021).

[28] Chen, X. & He, K. Exploring simple siamese representation learning. In *Proceedings of the IEEE/CVF conference on computer vision and pattern recognition*, pages 15750–15758, (2021).

[29] Ermolov, A., Siarohin, A., Sangineto, E. & Sebe, N. Whitening for self-supervised representation learning. In *International Conference on Machine Learning*, pages 3015–3024. PMLR, (2021).

[30] Zbontar, J., Jing, L., Misra, I., LeCun, Y. & Deny, S. Barlow twins: Self-supervised learning via redundancy reduction. In *International Conference on Machine Learning*, pages 12310–12320. PMLR, (2021).

[31] Bardes, A., Ponce, J. & LeCun, Y. Vicreg: Variance-invariance-covariance regularization for self-supervised learning. *arXiv preprint* arXiv:2105.04906, (2021).

[32] Hua, T. et al. On feature decorrelation in self-supervised learning. In *Proceedings of the IEEE/CVF International Conference on Computer Vision*, pages 9598–9608, (2021).

[33] Chang, W.-G., You, T., Seo, S., Kwak, S. & Han, B. Domain-specific batch normalization for unsupervised domain adaptation. In *Proceedings of the IEEE/CVF conference on Computer Vision and Pattern Recognition*, pages 7354–7362, (2019).

[34] Wolf, F. A., Angerer, P. & Theis, F. J. Scanpy: Large-scale single-cell gene expression data analysis. *Genome. Biol.* **19**, 1–5 (2018). 29409532 10.1186/s13059-017-1382-0 PMC5802054

[35] Huang, G., Liu, Z., Van Der Maaten, L. & Weinberger, K. Q. Densely connected convolutional networks. In *Proceedings of the IEEE conference on computer vision and pattern recognition*, pages 4700–4708, (2017).

[36] Hubert, L. & Arabie, P. *Comparing partitions. Journal of classification* **2**, 193–218 (1985).

[37] Pedregosa, F. et al. Scikit-learn: Machine learning in python. *J. Mach. Learn. Res.* **12**, 2825–2830 (2011).

[38] Rousseeuw, P. J. Silhouettes: A graphical aid to the interpretation and validation of cluster analysis. *J. Comput. Appl. Math.* **20**, 53–65 (1987).

[39] Büttner, M., Zhichao Miao, F., Wolf, A., Teichmann, S. A. & Theis, F. J. A test metric for assessing single-cell RNA-seq batch correction. *Nat. Methods.* **16**(1), 43–49 (2019). 30573817 10.1038/s41592-018-0254-1

[40] Xiong, L. et al. Online single-cell data integration through projecting heterogeneous datasets into a common cell-embedding space. *Nat. Commun.* **13**(1), 6118 (2022). 36253379 10.1038/s41467-022-33758-z PMC9574176

[41] McInnes, L., Healy, J. & Melville, J. Umap: Uniform manifold approximation and projection for dimension reduction. *arXiv preprint* arXiv:1802.03426, (2018).

[42] Domínguez Conde, C. et al. Cross-tissue immune cell analysis reveals tissue-specific features in humans. *Science* **376**(6594), eabl5197 (2022). 35549406 10.1126/science.abl5197 PMC7612735

[43] Zhao, B., Song, K., Wei, D.-Q., Xiong, Y. & Ding, J. Sccobra allows contrastive cell embedding learning with domain adaptation for single cell data integration and harmonization. *Commun. Biol.* **8**(1), 233 (2025). 39948393 10.1038/s42003-025-07692-x PMC11825689

[44] Lotfollahi, M., Naghipourfar, M., Theis, F. J. & Wolf, F. A. Conditional out-of-distribution generation for unpaired data using transfer VAE.. *Bioinformatics* **36**(Supplement_2), i610–i617 (2020). 33381839 10.1093/bioinformatics/btaa800

[45] Liu, J. et al. Jointly defining cell types from multiple single-cell datasets using Liger. *Nat. Protoc.* **15**(11), 3632–3662 (2020). 33046898 10.1038/s41596-020-0391-8 PMC8132955

[46] Hie, B., Bryson, B. & Berger, B. Efficient integration of heterogeneous single-cell transcriptomes using Scanorama. *Nat. Biotechnol.* **37**(6), 685–691 (2019). 31061482 10.1038/s41587-019-0113-3 PMC6551256

[47] Karl Pearson, F. R. S. & Liii. On lines and planes of closest fit to systems of points in space. *The London, Edinburgh, and Dublin Philosophical Magazine and Journal of Science* **2**(11), 559–572 (1901).

[48] Hao, Y. et al. Dictionary learning for integrative, multimodal and scalable single-cell analysis. *Nat. Biotechnol.* **42**, 293–304 (2023). 10.1038/s41587-023-01767-y PMC10928517 37231261

[49] Lu, L. & Welch, J. D. PyLiger: Scalable single-cell multi-omic data integration in Python. *Bioinformatics* **38**(10), 2946–2948 (2022). 35561174 10.1093/bioinformatics/btac190 PMC9306758

[50] Vieira, F. A. et al. A cellular census of human lungs identifies novel cell states in health and in asthma. *Nat. Med.* **25**(7), 1153–1163 (2019). 31209336 10.1038/s41591-019-0468-5

