# Supplementary material for: scDecorr: feature decorrelation based representation learning enables self-supervised alignment of multiple single-cell experiments
Source: Sci Rep. 2026 Apr 29;16:13782. doi: 10.1038/s41598-026-50586-z (PMC13128840; doi:10.1038/s41598-026-50586-z)
Supplement: Supplementary file 1 — Supplementary Information 1. [file 41598_2026_50586_MOESM1_ESM.pdf]

Supplementary File

scDecorr - Feature decorrelation based representation learning enables self-supervised alignment of multiple single-cell experiments

Ritabrata Sanyal, Yang Xu, Hyojin Kim, Rafael Kramann, Sikander Hayat

1 Supplementary Tables

Table S1: Datasets details - overview of datasets used in this study

Dataset	Batches (Integration)[1]	# Batches (Integration)	Batches (Label Transfer)[2]	# Batches (Label Transfer)	# Cells	# Cell Types	Shared Cell Types (%) (Atleast 2 Batches)[3]	Shared Cell Types (%) (All Batches)[3]
Crosstissue Immune [1]	5v1, 5v2, 3	3	5v1, 5v2, 3	3	216611	18	100 %	100 %
Human Immune [2]	10X, Freytag, Oetjen_A, Oetjen_P, Oetjen_U, Sun_sample1_CS, Sun_sample2_KC, Sun_sample3_TB, Sun_sample4_TC, Villani	10	10X, Freytag, Oetjen, Sun, Villani	5	33506	16	75 %	19 %
Human Lung [2]	1–6, A1–A6, B1–B4	16	1–6 (10X_Transplant), A1–A6 (10X_Biopsy), B1–B4 (DropSeq_Transplant)	3	32472	17	65.00 %	53 %
Human Pancreas [2]	celseq, celseq2, fluidigmc1, inDrop1, inDrop2, inDrop3, inDrop4, smarter, smartseq2	9	celseq, celseq2, fluidigmc1, inDrop, smarter, smartseq2	6	16382	14	93 %	29 %
Tabula Muris [3]	Droplet, FACS	2	Droplet, FACS	2	67354	28	93 %	93 %

[1] Data integration is performed on individual donor batches
[2] Label transfer is performed on distinct experimental batches (group of donor batches)
[3] Shared cell types are computed on the label transfer batches

Table S2: Best training configurations of scDecorr for every dataset

	Dataset	Mini-Batch Size	Encoder (# layers)	Projector (layers)	References
1	Cross-tissue Immune	2048	21	1024-1024-1024	[1]
2	Human Immune	512	11	512-512-512	[2]
3	Human Lungs	512	11	512-512-512	[2]
4	Human Pancreas	512	11	512-512-512	[2]
5	Tabula Muris	2048	21	1024-1024-1024	[3]

S We also include the following tables as excel spreadsheets along with our manuscript:

- (Table S3) **Dataset Details**: Detailed overview of all datasets along with cell-type distributions of each dataset. Batch specific cell-types of each dataset are also included.
- (Table S4) **Individual Data Integration Scores**: This spreadsheet contains two sheets namely,
  - Raw Scores (Table S4.1): Raw data integration scores such as ARI, NMI, CellType ASW, Batch ASW, Batch Entropy, Graph Connectivity, Overcorrection.
  - Normalised Scores (Table S4.2): Corresponding min-max normalised scores. Normalised scores are used to compute aggregated metrics such as S_{bio}, S_{batch}, S_{oc}, $S_{data\_integration}$
- (Table S5) **Aggregated Data Integration Scores**: This spreadsheet contains two sheets namely,
  - All Scores (Table S5.1): Aggregated data integration scores such as S_{bio}, S_{batch}, S_{oc}, $S_{data\_integration}$ for all datasets
  - Dataset Average Scores (Table S5.2): Aggregated data integration scores averaged across datasets. This is used to rank methods across all datasets.
- (Table S6) **Individual Label Transfer Scores**: This spreadsheet contains two sheets namely,
  - Raw Scores (Table S6.1): Raw label transfer scores such as batch-wise ARI, NMI, CellType ASW, F1, Accuracy.
  - Normalised Scores (Table S6.2): Corresponding min-max normalised scores. Nor-

malised scores are used to compute aggregated metrics such as S_{bw_bio}, $S_{classify}$, $S_{label\_transfer}$.

- (Table S7) **Aggregated Label Transfer Scores**: This spreadsheet contains two sheets namely,
  - All Scores (Table S7.1): Aggregated label transfer scores such as S_{bw_bio}, $S_{classify}$, $S_{label\_transfer}$ for all batches in all datasets
  - Batch Average Scores (Table S7.2): Batch averaged aggregated label transfer scores for all datasets.
  - Dataset Average Scores (Table 7.3): Aggregated label transfer scores averaged across datasets. This is used to rank methods across all datasets.
- (Table S8) **Cluster Resolutions**: Best Leiden clustering resolutions of each method and number of clusters detected.
- (Table S9) **Ablation Studies**: This spreadsheet contains two sheets namely,
  - Data Augmentations (Table S9.1): Ablations of different types of data augmentations.
  - Hyperparameter Ablations (Table S9.2): Ablations of different training configurations
- (Table S10) **Human Immune Marker Genes scDecorr**: Cluster marker genes of human immune dataset as obtained by scDecorr

2 Ablation Studies

2.1 Data Augmentations

We explore various data augmentation techniques, including random shuffle, random dropouts, random swap, and the addition of Gaussian noise, along with their combinations, to identify the most effective augmentation transformations. These experiments were conducted on the cross-tissue immune dataset, with the findings detailed in Table S9.1. Some of the transformations used were sourced from [4]. Below is a concise overview of the transformations evaluated. Every transformation was applied with probability of 0.5.

- Random Shuffle: Randomly shuffle $k\%$ of input gene expression values.
- Random Zero: Randomly dropout $k\%$ of input gene expression values.
- Random Swap: Randomly swap $k\%$ of input gene expression values by arbitrarily constructing pairs. This transformation was sourced from [4].
- Gaussian Noise: Randomly corrupt $k\%$ of input gene expression values with Gaussian Noise. This transformation was sourced from [4].

According to Table S9.1, the combination of random shuffle ($k = 10\%$) and random zero ($k = 20\%$) transformations yields the best results.

2.2 Training Configurations

In order to determine the optimal training configurations for scDecorr in each dataset, we conduct a systematic hyperparameter search across various key hyperparameters. These include mini-batch size, the number of layers in the encoder, the dimension of the decoder layer, the number of selected highly variable genes (HVGs), and the lambda parameter in the

loss function. Additionally, we present results without the use of DSBN layers to highlight the importance of using DSBN layers for batch correction. To avoid an overwhelming number of hyperparameter combinations, we limit the selection to a few values for each hyperparameter, altering only one at a time while keeping the others at their default settings. For this analysis, we choose 2 small datasets ($\leq 50k$ cells), namely Human Lung, and Human Pancreas, and 2 large datasets ($> 50k$ cells) namely Tabula Muris, and Cross-Tissue Immune datasets. The results are presented in Table S9.2, where we provide the ARI and batch entropy scores for each hyperparameter configuration. Additionally, we include the difference between the scores of a specific hyperparameter setting and the default setting scores to illustrate the sensitivity of each hyperparameter. From Table S9.2, we observe that the optimal lambda value across all datasets is 0.05. For smaller datasets ($\leq 50k$ cells) like human lung, and human pancreas, a mini-batch size of 512, 11 encoder layers, and decoder layer dimensions of 512 are most effective. For larger datasets ($\geq 50k$ cells) such as Tabula Muris and cross-tissue immune atlas, a mini-batch size of 2048, 21 encoder layers, and decoder layer dimensions of 1024 are optimal. Additionally, we can observe that without using DSBN layers, batch correction performance degrades drastically across all datasets, emphasizing the importance of using DSBN to facilitate proper data integration.

3 Biological interpretability and rare Platelet population recovery using scDecorr

To evaluate whether scDecorr preserves and enhances biological interpretability beyond numerical integration metrics, we applied scDecorr to a human immune single-cell RNA-seq dataset with existing author-provided annotations and multiple sequencing batches. In the

integrated embedding produced by scDecorr, major immune lineages segregated into well-defined manifolds, including CD14+ monocytes, CD16+ monocytes, plasmacytoid dendritic cells, hematopoietic stem and progenitor cell (HSPC), B cells, T cells, natural killer (NK) cells, erythroid and dendritic lineage cells (See Fig. S1A, B, C).

To systematically assess biological coherence of the inferred clusters, we computed the marker genes of each cluster using differential expression and compared the obtained markers against known reference markers of immune cell lineages using over-representation analysis (ORA). The reference markers were collected from the Azimuth, CellTypist and CellMarkers databases. ORA enrichment patterns were consistent with canonical immune programs and supported scDecorr cluster identities (Fig.S1 B, E). Moreover, scDecorr-derived annotation broadly agreed with the original author-provided annotation and preserved biologically meaningful cell identity signals (Fig.S1 K, M, N). Marker gene visualizations further confirmed that clusters corresponded to established lineage signatures rather than batch-dependent groupings (Fig.S1 F, G, H, I, J).

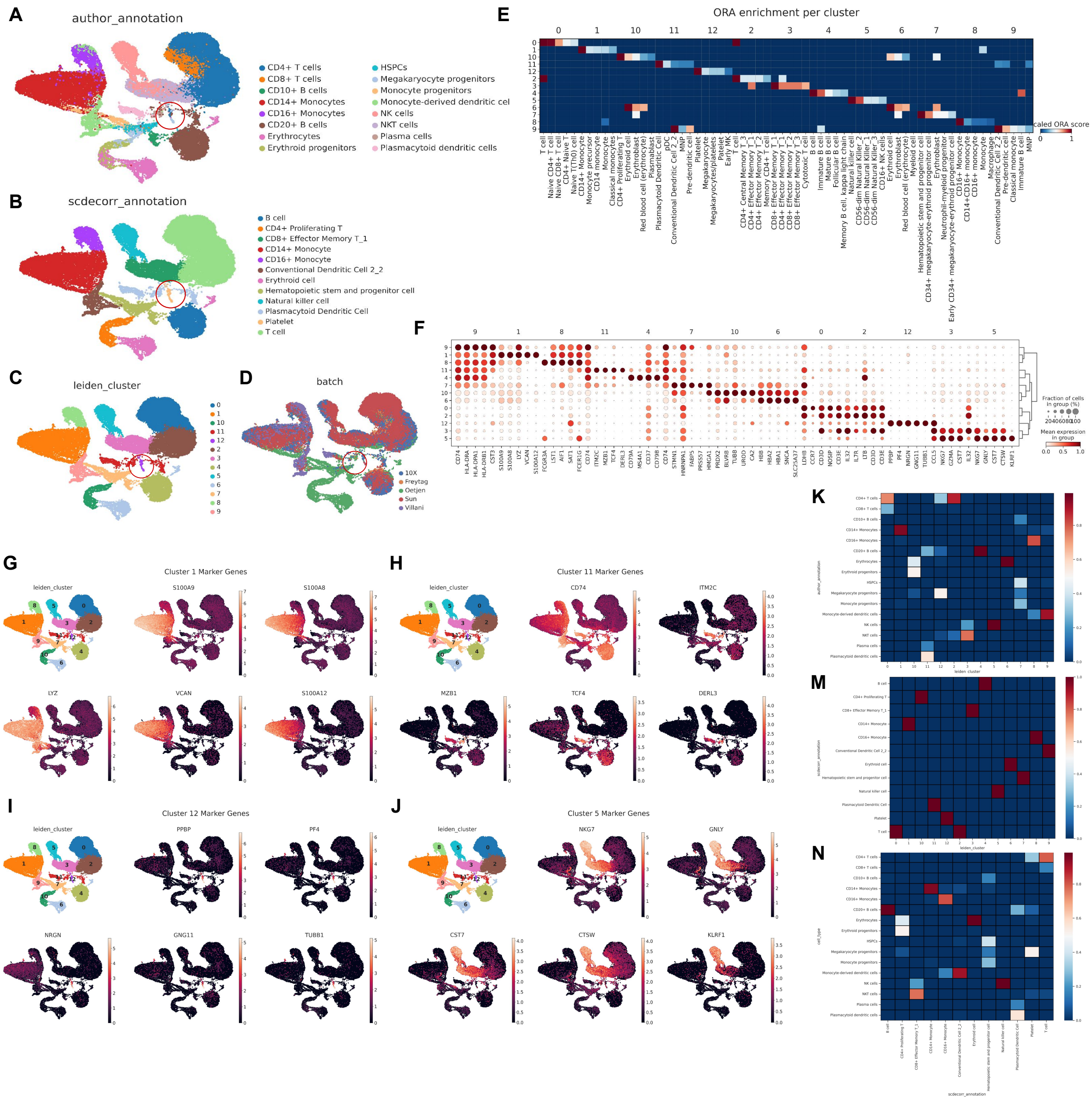

Figure S1: Annotating human immune dataset using scDecorr embeddings and marker gene analysis. A, B, C, D. UMAP visualization of scDecorr embeddings labeled with author provided cell-type annotations (A), scDecorr-derived cluster annotations (B), cluster labels (C), and batch names (D) respectively, E. Heatmap visualizing top enriched cell-types for each cluster (based on marker gene over-representation analysis). F. Dotplot showing top 5 marker genes per cluster. G, H, I, J. UMAPs visualizing top 5 marker genes expression for cluster 1, 11, 12, 5 respectively. K, M. Heatmaps showing author annotated (K) and scdecorr annotated cell-types (M) per cluster. N. Heatmap showing overlap between author annotated and scdecorr annotated cell-types.

Moreover, scDecorr resolved a small but distinct cluster (Cluster 12) characterized by a highly specific platelet transcriptional program (Fig. S2 A). This population was previously annotated by the dataset authors as a mixed group containing predominantly megakaryocyte progenitors with additional contamination from CD4+ T cells, CD20+ B cells and NKT/T cells (Fig. S2 L). In contrast, scDecorr yielded a compact cluster with strong and selective expression of canonical platelet markers including *PPBP, PF4, PF4V1, GP9, ITGA2B, ITGB3, GP1BA, TUBB1, RGS18, NRGN, and GNG11* (Fig. S2 A, B, C, M). This platelet signature was concentrated in cluster 12 and largely absent from other immune clusters (Fig.S2 B, C, M).

To exclude that this cluster represents technical mixtures (doublets) or platelet–leukocyte aggregates, we assessed expression of canonical leukocyte lineage markers. Cluster 12 showed minimal expression of pan-leukocyte and lineage genes including *PTPRC*, T cell markers (*CD3D, CD3E*), myeloid/monocyte markers (*LST1, LYZ*), B cell markers (*MS4A1*), and natural killer/cytotoxic markers (*NKG7, GNLY*), compared to their strong expression in the corresponding leukocyte clusters (Fig. S2 B, C, N). In addition, a module score comparison demonstrated that cluster 12 has a strong platelet signature while lacking enrichment for leukocyte programs (Fig. S2 E, F, G). Together, this supports that cluster 12 represents a platelet-like population rather than a cluster dominated by leukocyte doublets.

Since platelets originate from megakaryocytes, we further tested whether the cluster could reflect megakaryocyte progenitors rather than platelet-like droplets. Cluster 12 did not show strong enrichment of megakaryocyte progenitor transcription factors such as *GATA1, GATA2, TAL1, NFE2, LMO2, and ETS1*, and it also lacked enrichment of proliferation-associated cell cycle markers (e.g. *MKI67, TOP2A, STMN1*) (Fig. S2 D, N). This indicates that the

population is unlikely to represent cycling megakaryocyte progenitors and is more consistent with a matured platelet transcriptional program.

Finally, we assessed basic RNA complexity metrics. Cells in cluster 12 showed reduced library complexity compared to typical leukocytes, with fewer detected genes and lower UMI, consistent with the low RNA content expected for platelet-derived droplets (Fig. S2 H, I). To assess whether this population was batch-specific, we quantified its abundance across sequencing batches. Cluster 12 was detected across multiple batches, with low but consistent abundance, and its batch composition was not dominated by a single dataset (Fig. S2 J, K). Together, these results indicate that the platelet-like cluster recovered by scDecorr is reproducible across batches and is unlikely to reflect a batch-driven artifact. Moreover, alternative integration approaches such as scVI and scCobra did not recover an equivalently well-resolved platelet cluster in the integrated embedding. In these embeddings, the platelet-like cells remained largely obscured into nearby populations, making marker-based annotation less straightforward (Fig. S3). This highlights that scDecorr can improve biological interpretability by enhancing separation of rare populations that may be difficult to resolve using existing integration methods. In summary, this analysis demonstrates that scDecorr not only preserves major immune cell identities in integrated datasets, but can also recover rare and biologically interpretable populations that were not clearly separated in the original annotation.

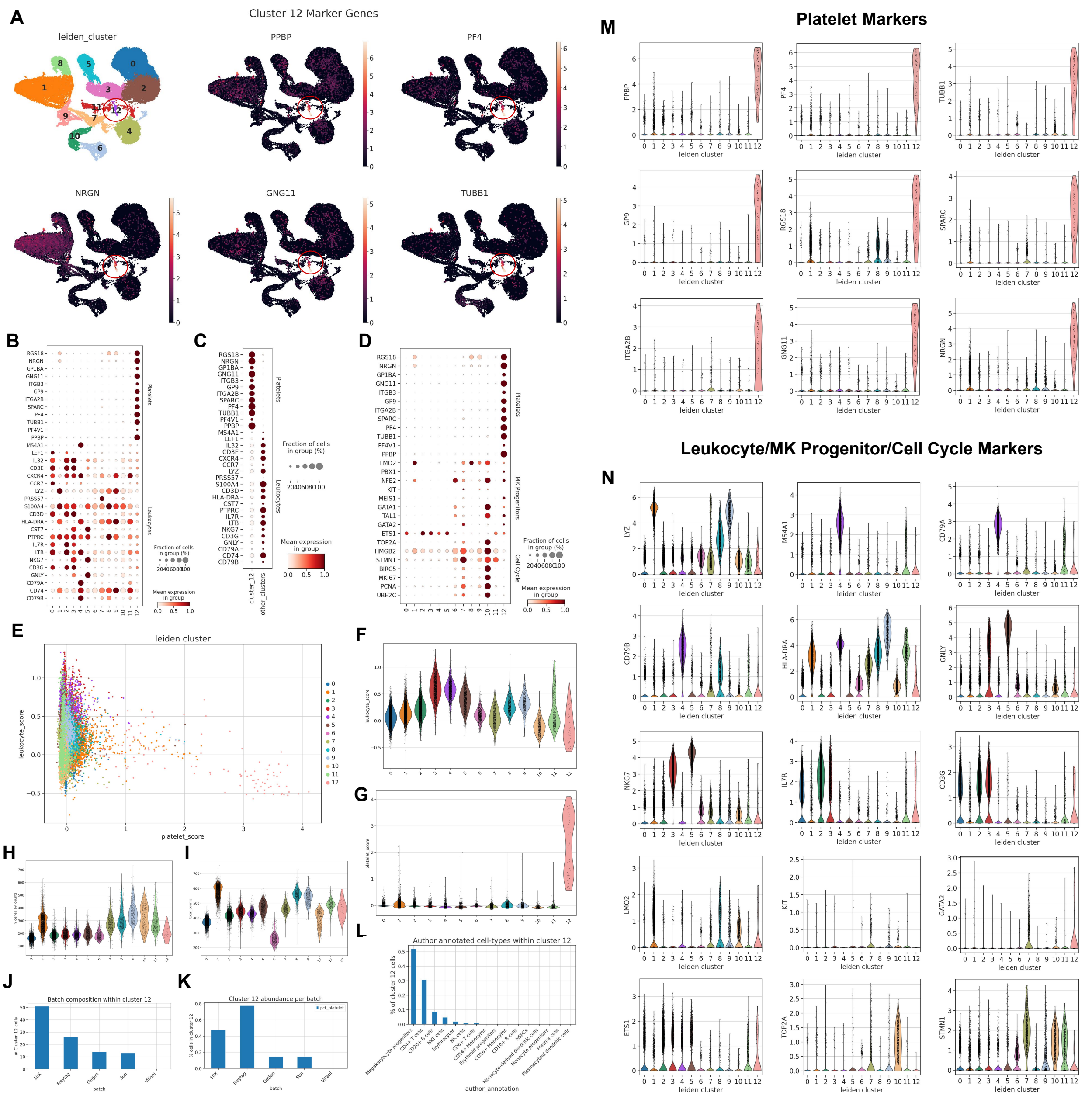

Figure S2: Rare platelet cell-type discovery in human immune dataset based on scDecorr embeddings, A. UMAPs visualizing top 5 marker genes expression for Platelet cluster (Cluster 12 marked in UMAPs). B. C. D. Dotplots visualizing enrichment of platelet and leukocyte markers in all clusters (B), enrichment of platelet and leukocyte markers in cluster 12 vs other clusters (C), enrichment of platelet, MK progenitor and cell-cycle markers in all clusters (D), E. Scatterplot showing leukocyte vs platelet module scores in each cluster, F,G. Violin plots showing leukocyte and platelet module scores distribution in each cluster, H, I. Violin plots showing count of expressed genes (H) and total UMI (I) in each cluster, J, K. Barplots showing batch composition in cluster 12 (J), and cluster 12 abundance per batch respectively (K), L. Barplots showing author-annotated cell-types in cluster 12, M, N. Violin plots visualizing Platelet markers expression in each cluster (M), and Leukocyte, MK Progenitor, and cell-cycle markers expression in each cluster (N).

4 Supplementary Figures

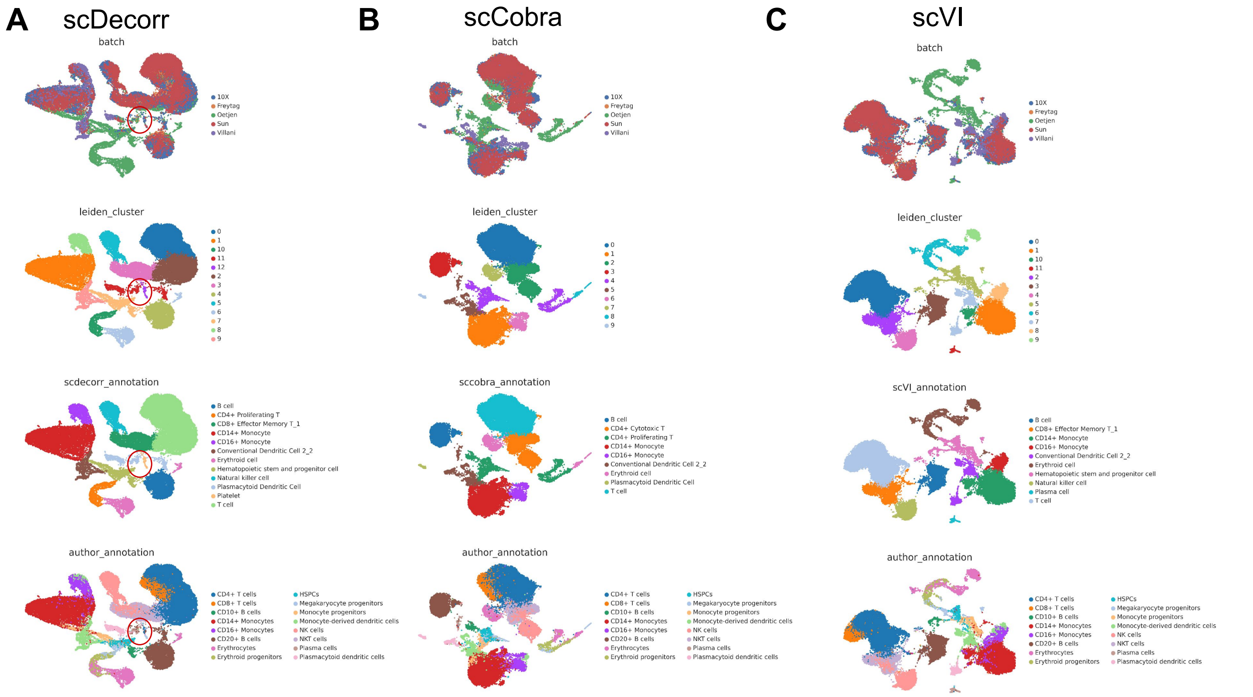

Figure S3: Cell-type annotations using A. scDecorr B. scCobra, and C. scVI embeddings. Author annotations and corresponding cluster labels are provided for clarity.

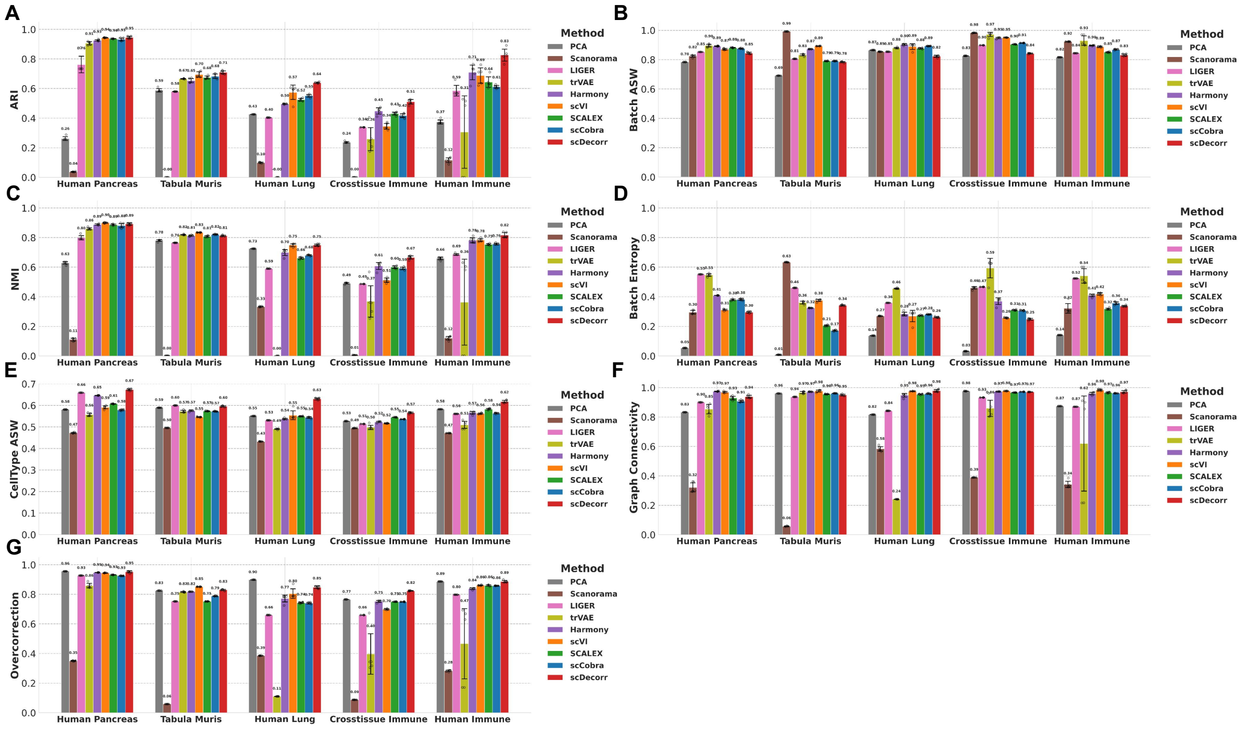

(1) Data integration scores

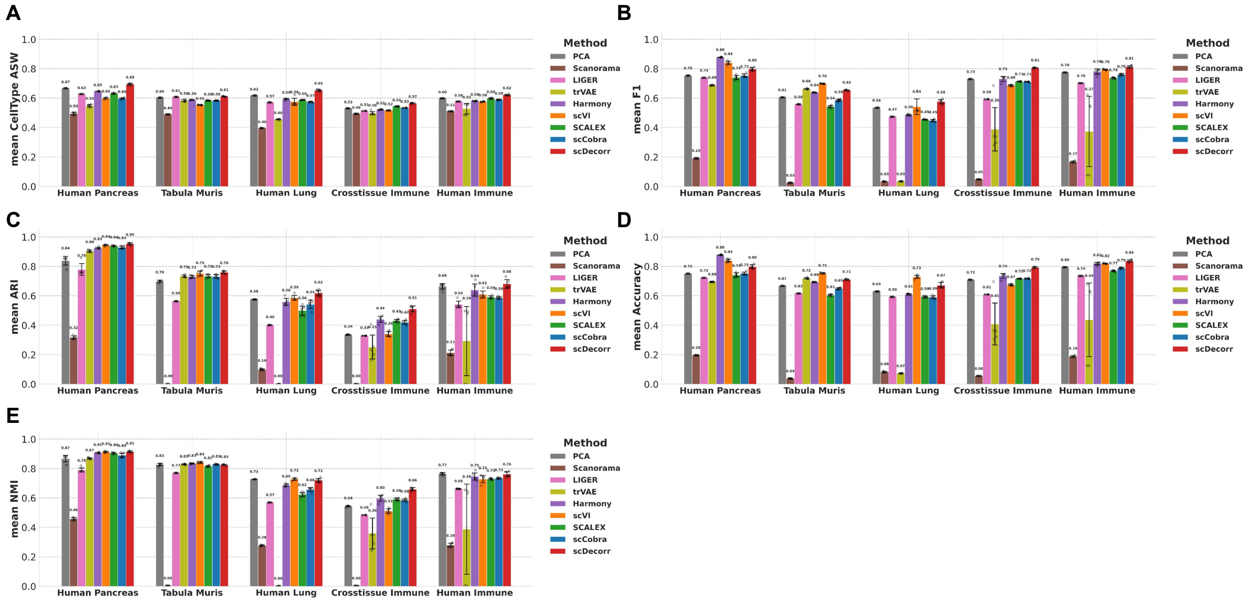

(2) Label transfer scores

Figure S4: Raw data integration (1) and label transfer (2) scores of all benchmark methods across all datasets visualized as barplots. Error bars indicate variance across 5 independent seed runs.

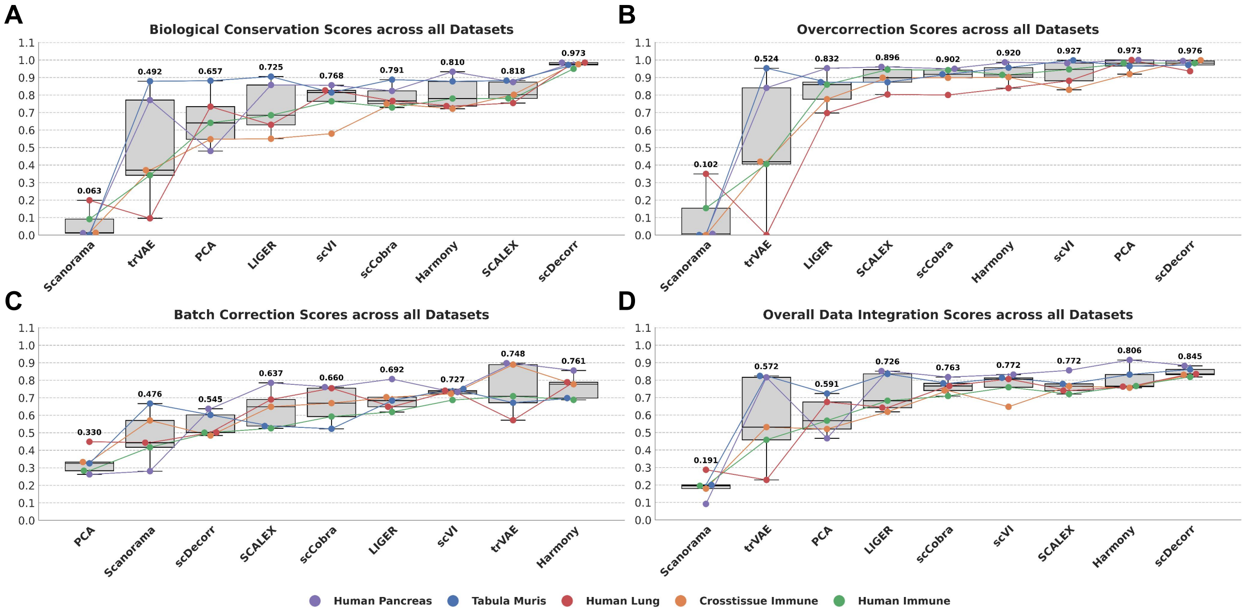

(1) Overall Data Integration Scores

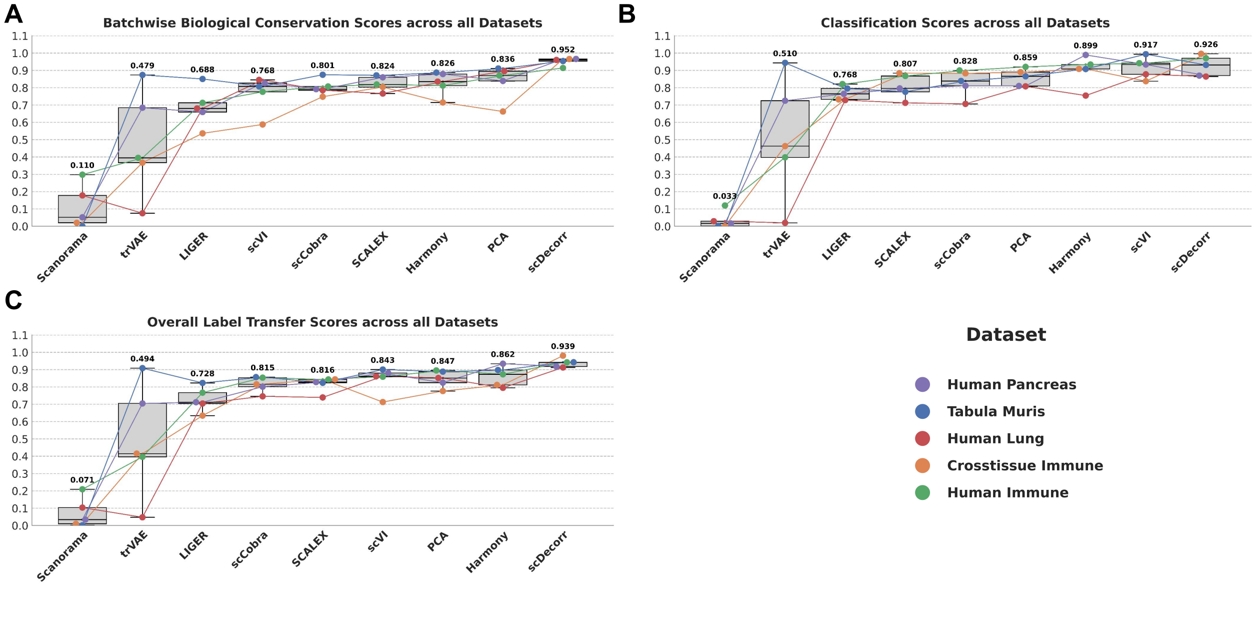

(2) Overall Label Transfer Scores

Figure S5: Aggregated data integration (1) and label transfer (2) scores of all benchmark methods visualized as boxplots across datasets. Boxplots are ordered from lowest to highest scoring methods in an ascending order with mean normalized scores across datasets annotated on top of the boxes.

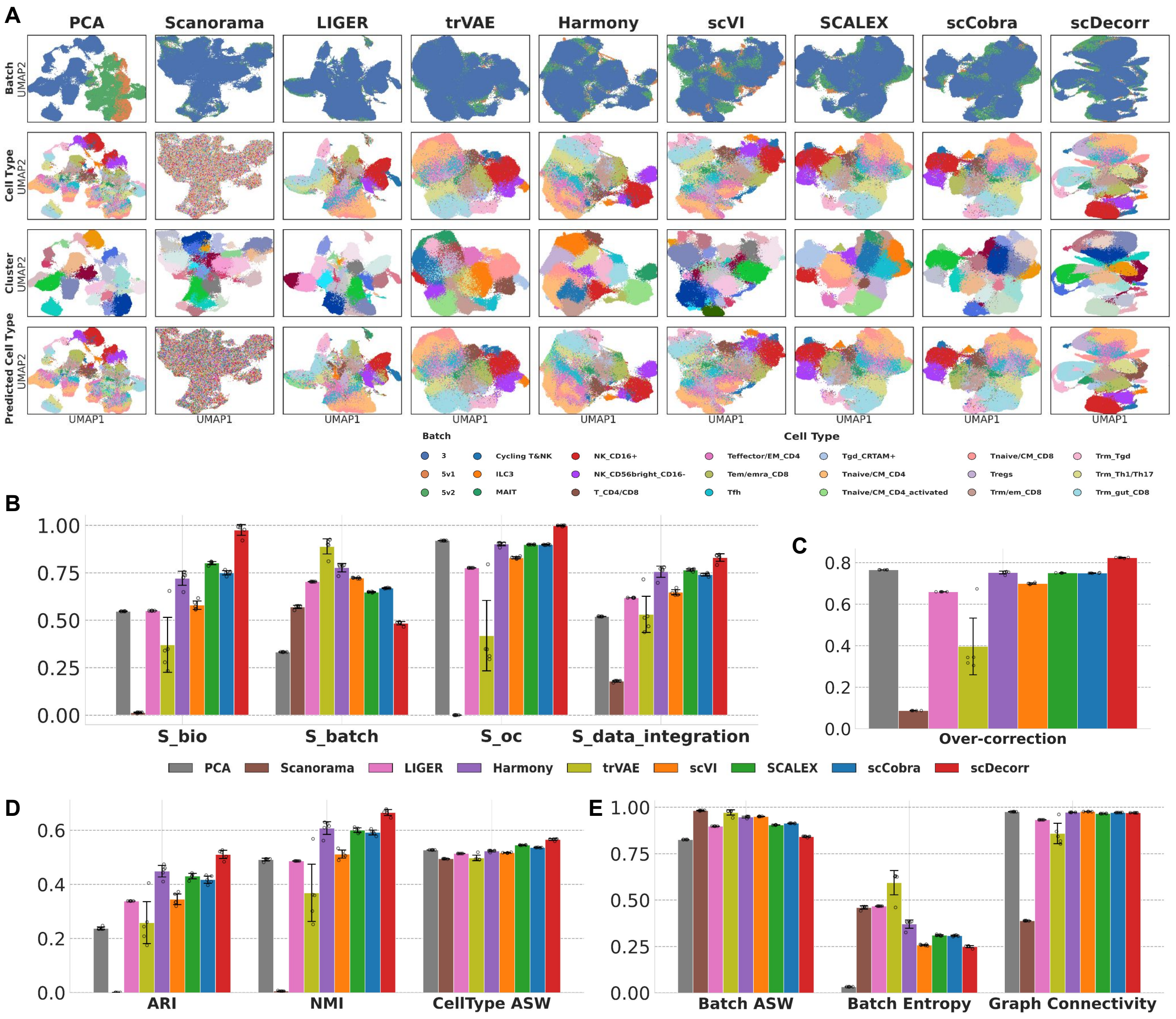

Figure S6: Data integration results on the cross-tissue immune dataset. A. UMAP visualizations of benchmark method embeddings annotated with batch, author annotated cell-type, cluster and predicted cell-type labels (using inter-batch label transfer), B. Aggregated data integration scores of all benchmark methods visualized as barplots. Barplots visualizing raw over-correction (C), biological conservation (D) and batch correction (E) scores of all benchmark methods respectively.

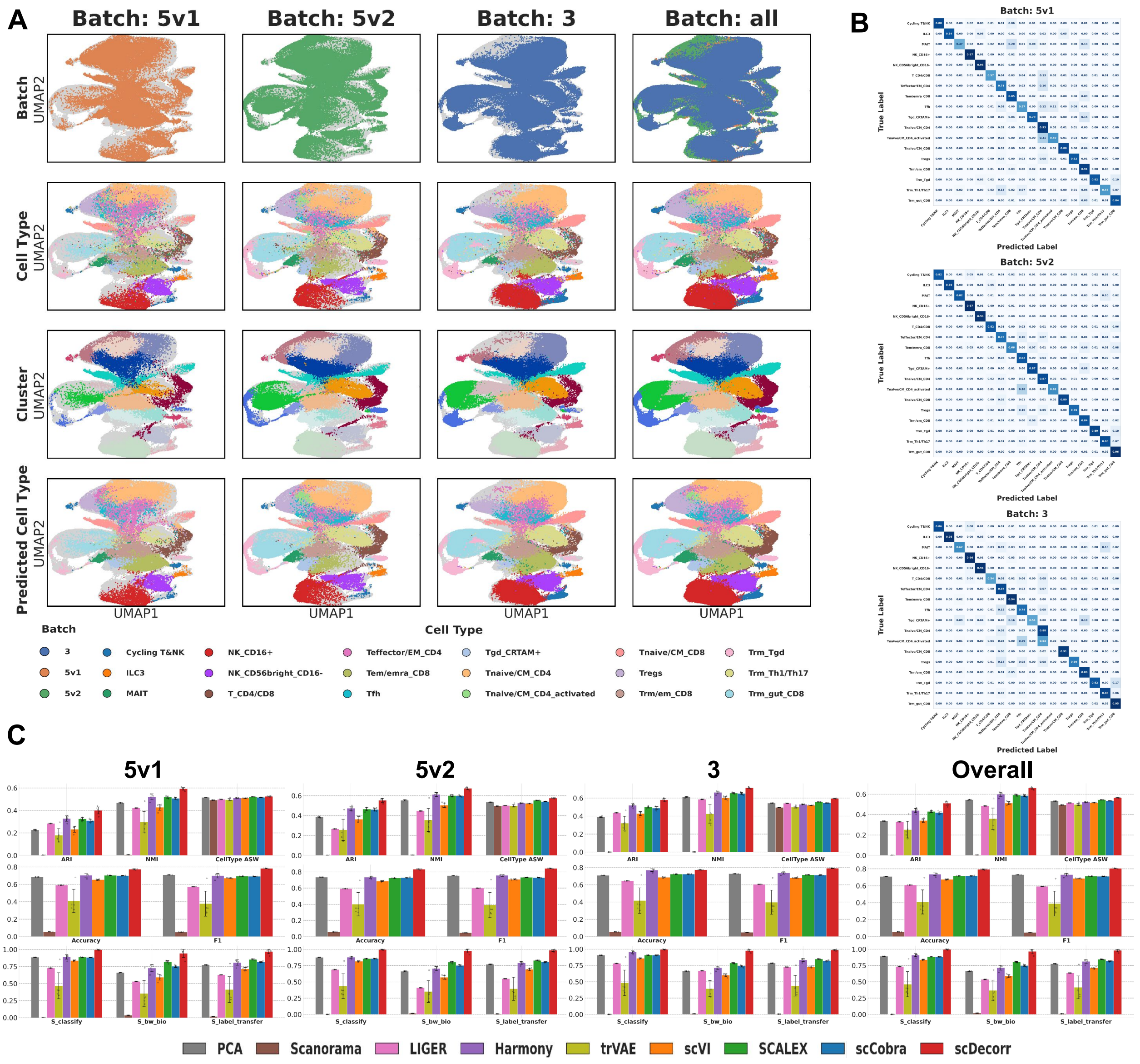

Figure S7: Label transfer results on the cross-tissue immune dataset. A. UMAPs visualizing batch-wise predicted cell-types by scDecorr using other batches as reference. The plots also include batch-labels, author annotated cell-types, and cluster labels. B. Confusion matrices showing TPR (recall) of label transfer classification performance in every batch. C. Barplots showing batch-wise label transfer performance scores achieved by all benchmark methods. Error bars indicate variance of scores across 5 independent runs.

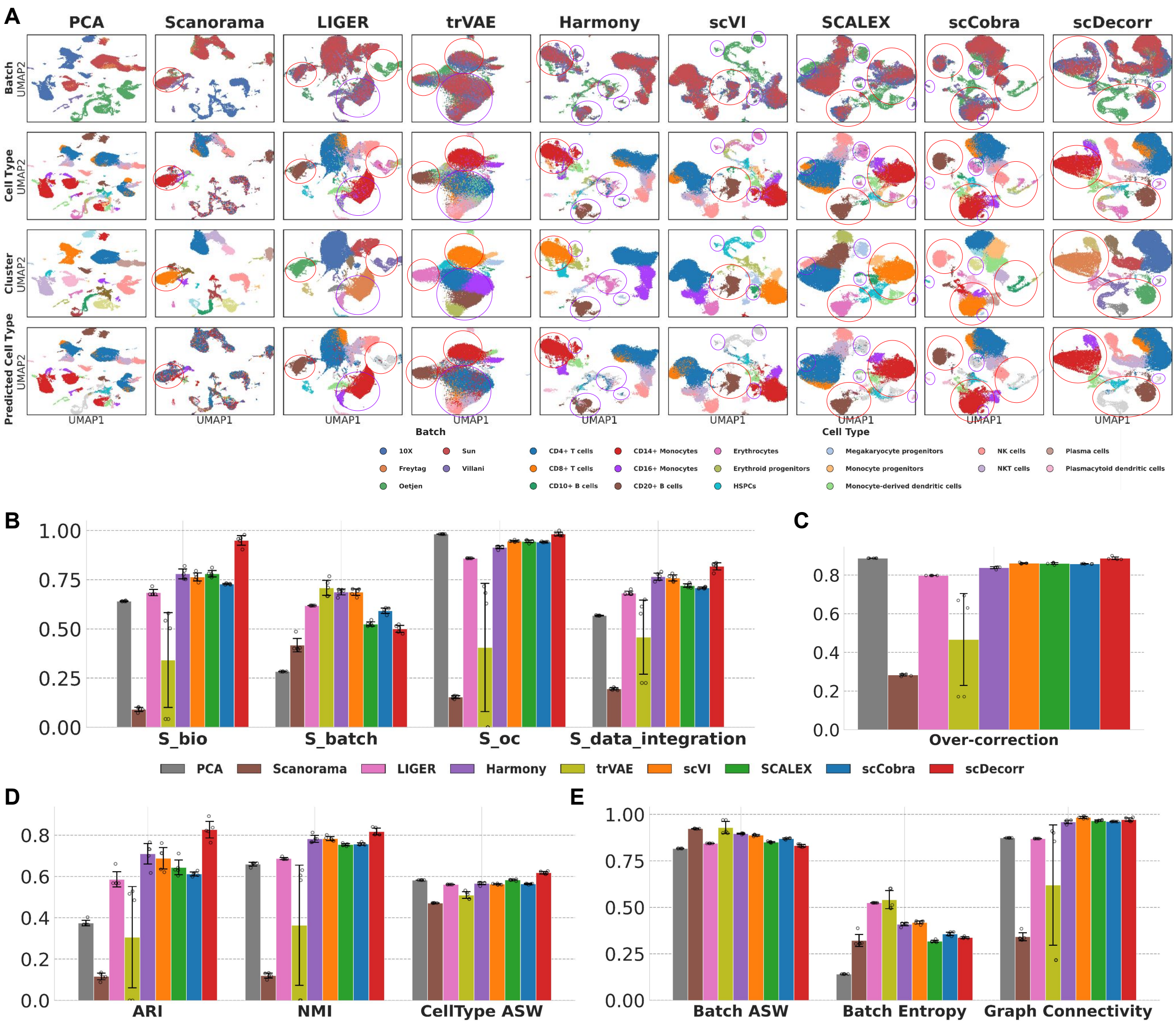

Figure S8: Data integration results on the human immune dataset. A. UMAP visualizations of benchmark method embeddings annotated with batch, author annotated cell-type, cluster and predicted cell-type labels (using inter-batch label transfer). Regions where methods perform well are shown in red, and those where they perform poorly are shown in violet, B. Aggregated data integration scores of all benchmark methods visualized as barplots. Barplots visualizing raw over-correction (C), biological conservation (D) and batch correction (E) scores of all benchmark methods respectively.

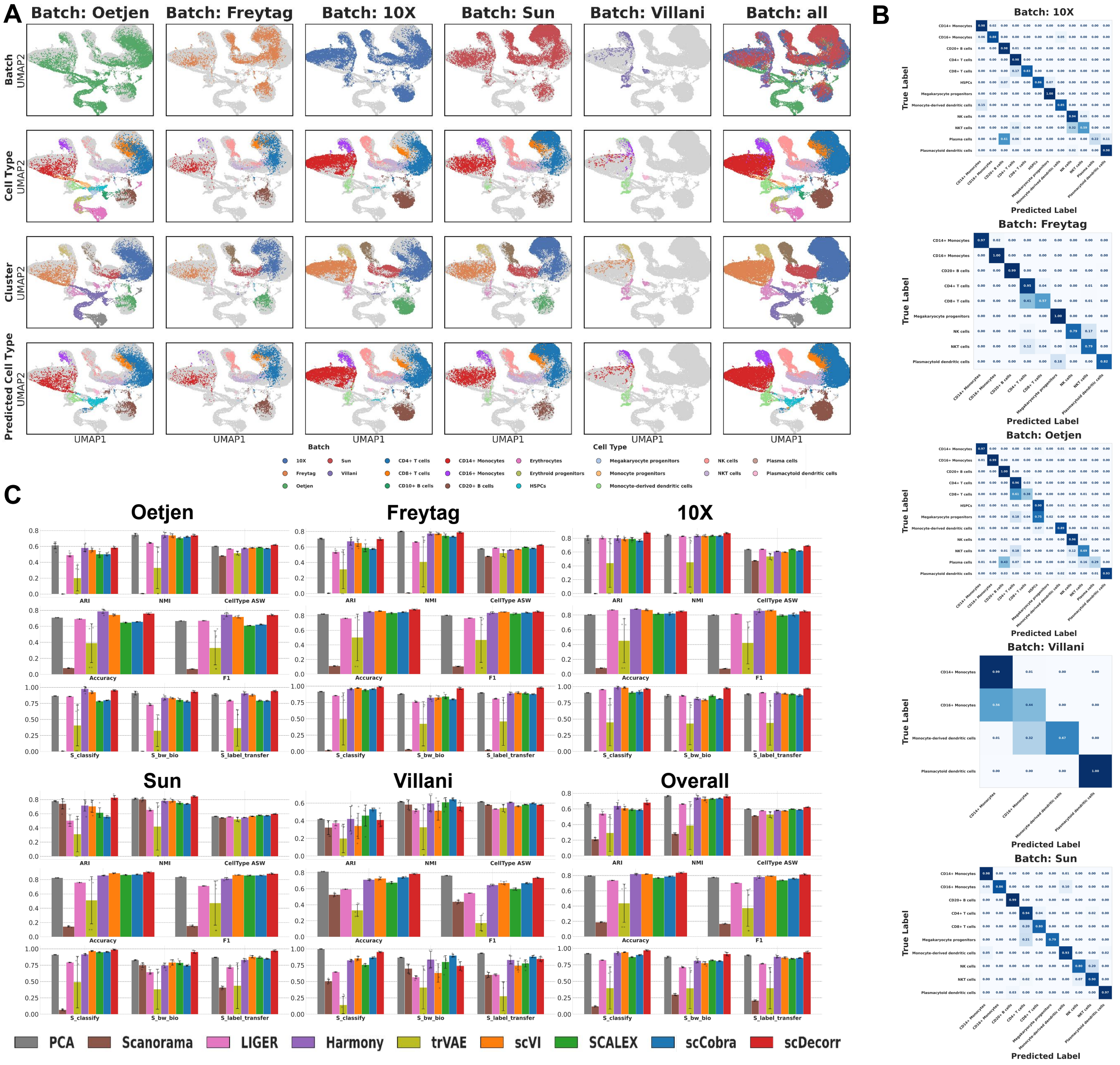

Figure S9: Label transfer results on the human immune dataset. A. UMAPs visualizing batch-wise predicted cell-types by scDecorr using other batches as reference. The plots also include batch-labels, author annotated cell-types, and cluster labels. B. Confusion matrices showing TPR (recall) of label transfer classification performance in every batch. C. Barplots showing batch-wise label transfer performance scores achieved by all benchmark methods. Error bars indicate variance of scores across 5 independent runs.

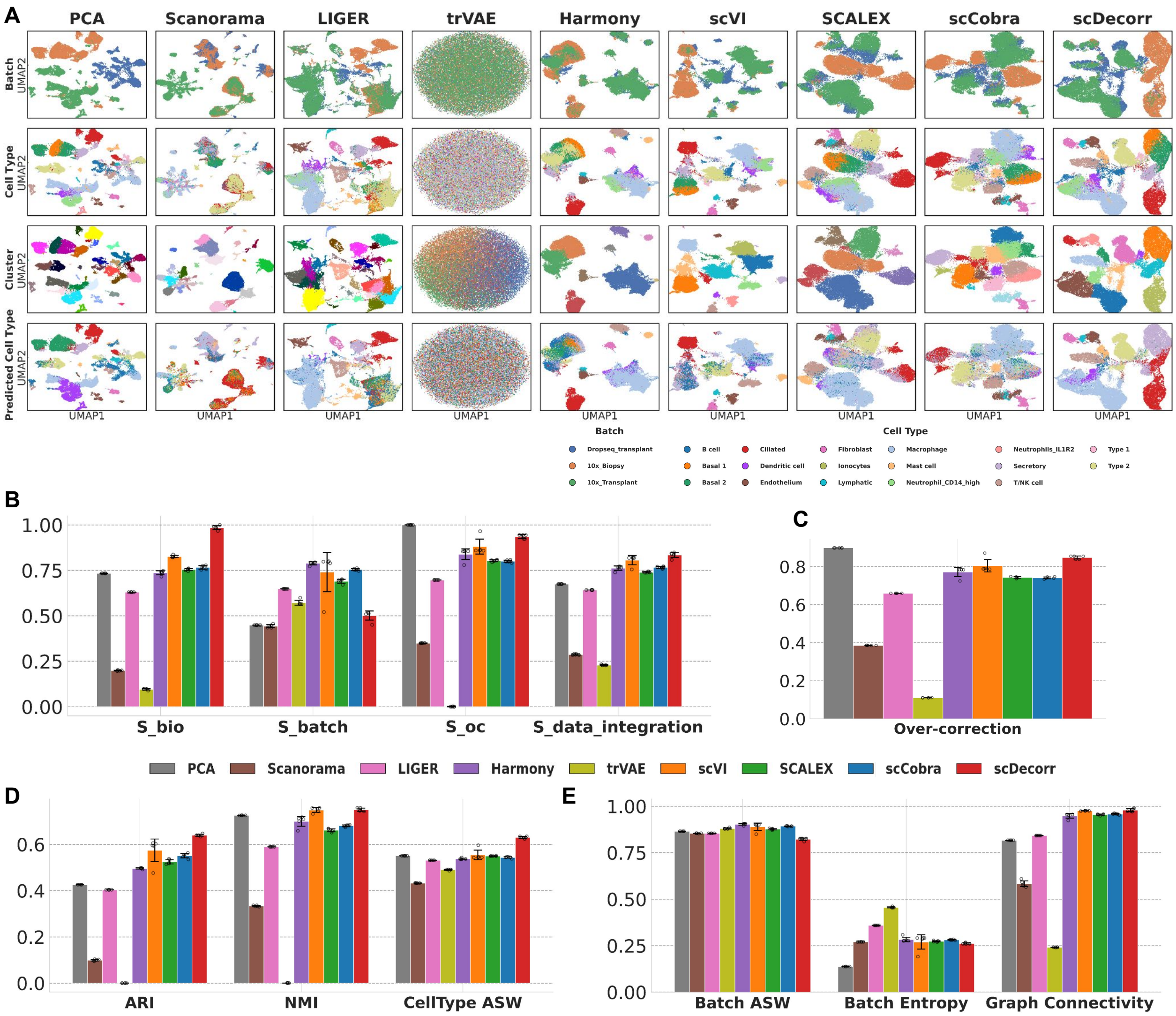

Figure S10: Data integration results on the human lung dataset. A. UMAP visualizations of benchmark method embeddings annotated with batch, author annotated cell-type, cluster and predicted cell-type labels (using inter-batch label transfer), B. Aggregated data integration scores of all benchmark methods visualized as barplots. Barplots visualizing raw over-correction (C), biological conservation (D) and batch correction (E) scores of all benchmark methods respectively.

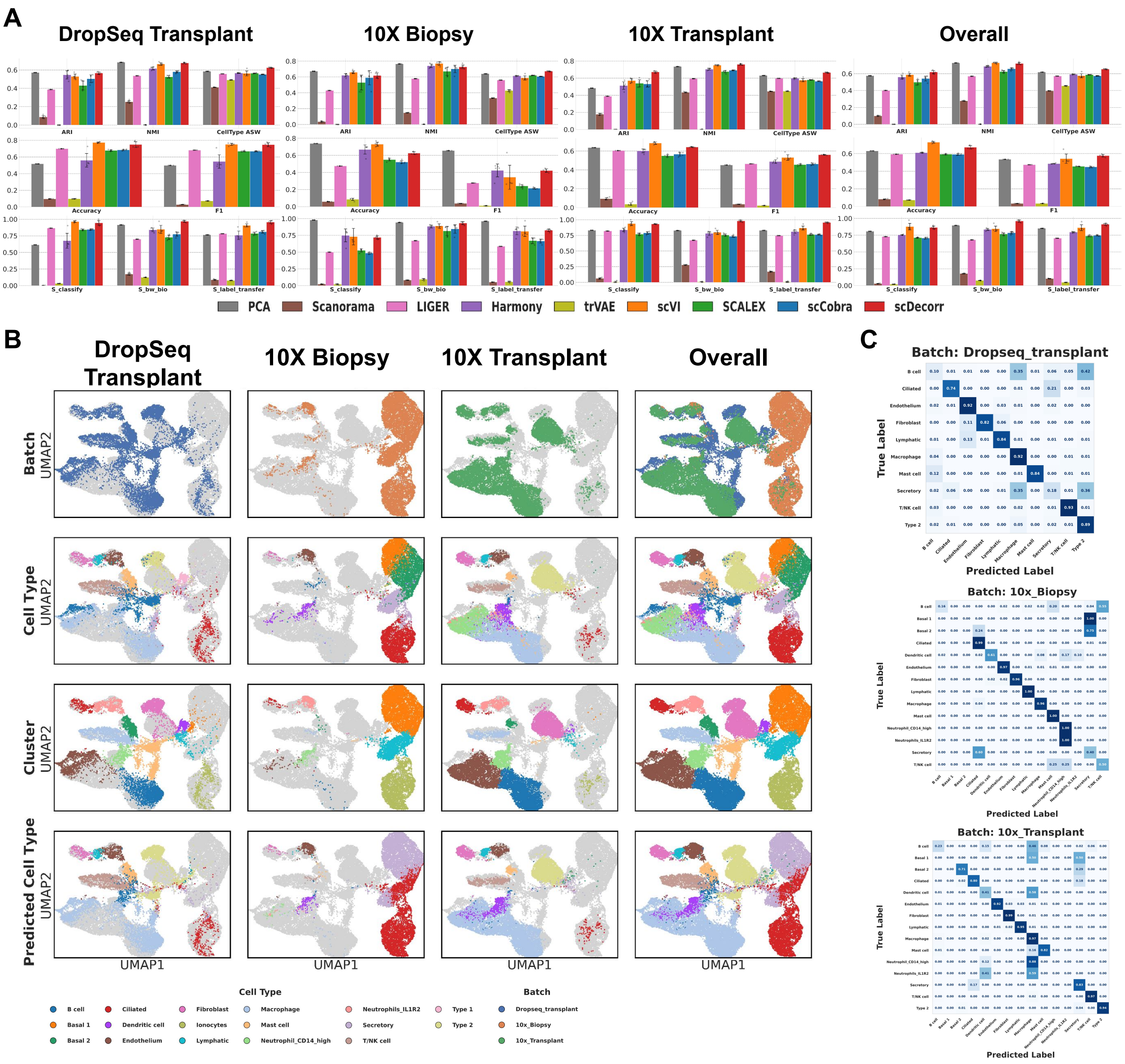

Figure S11: Label transfer results on the human lung dataset. A. UMAPs visualizing batch-wise predicted cell-types by scDecorr using other batches as reference. The plots also include batch-labels, author annotated cell-types, and cluster labels. B. Confusion matrices showing TPR (recall) of label transfer classification performance in every batch. C. Barplots showing batch-wise label transfer performance scores achieved by all benchmark methods. Error bars indicate variance of scores across 5 independent runs.

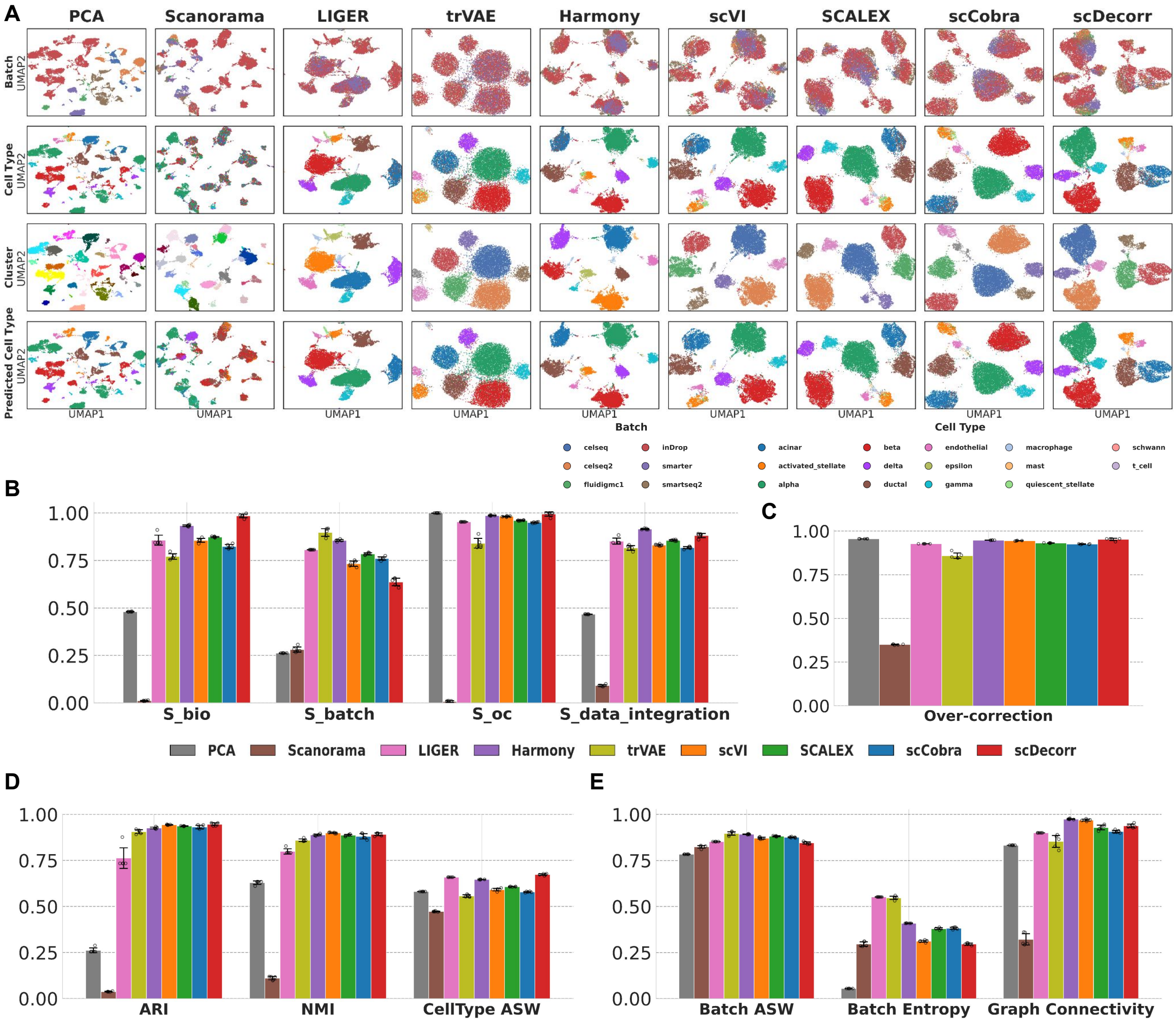

Figure S12: Data integration results on the human pancreas dataset. A. UMAP visualizations of benchmark method embeddings annotated with batch, author annotated cell-type, cluster and predicted cell-type labels (using inter-batch label transfer), B. Aggregated data integration scores of all benchmark methods visualized as barplots. Barplots visualizing raw over-correction (C), biological conservation (D) and batch correction (E) scores of all benchmark methods respectively.

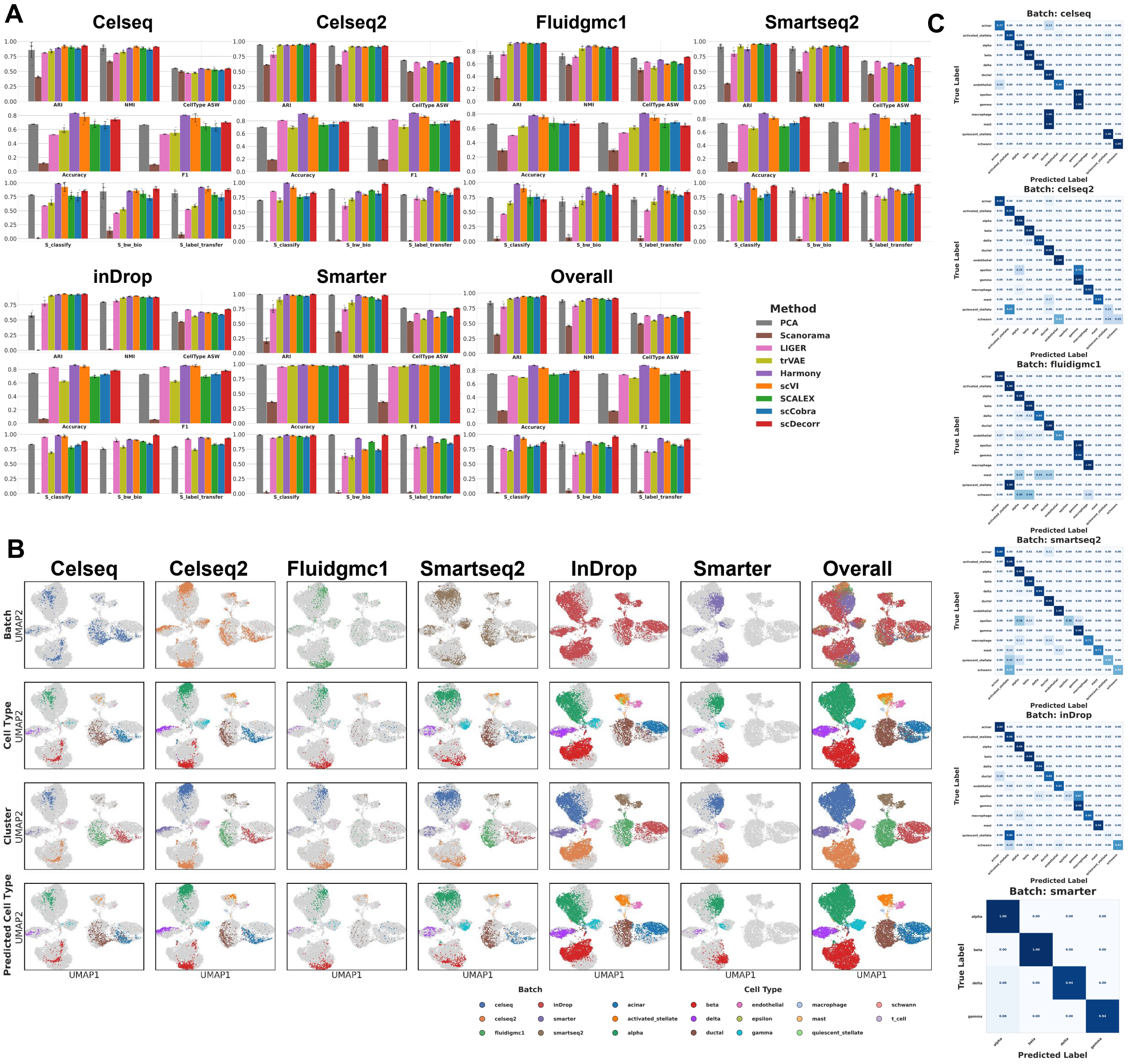

Figure S13: Label transfer results on the human pancreas dataset. A. UMAPs visualizing batch-wise predicted cell-types by scDecorr using other batches as reference. The plots also include batch-labels, author annotated cell-types, and cluster labels. B. Confusion matrices showing TPR (recall) of label transfer classification performance in every batch. C. Barplots showing batch-wise label transfer performance scores achieved by all benchmark methods. Error bars indicate variance of scores across 5 independent runs.

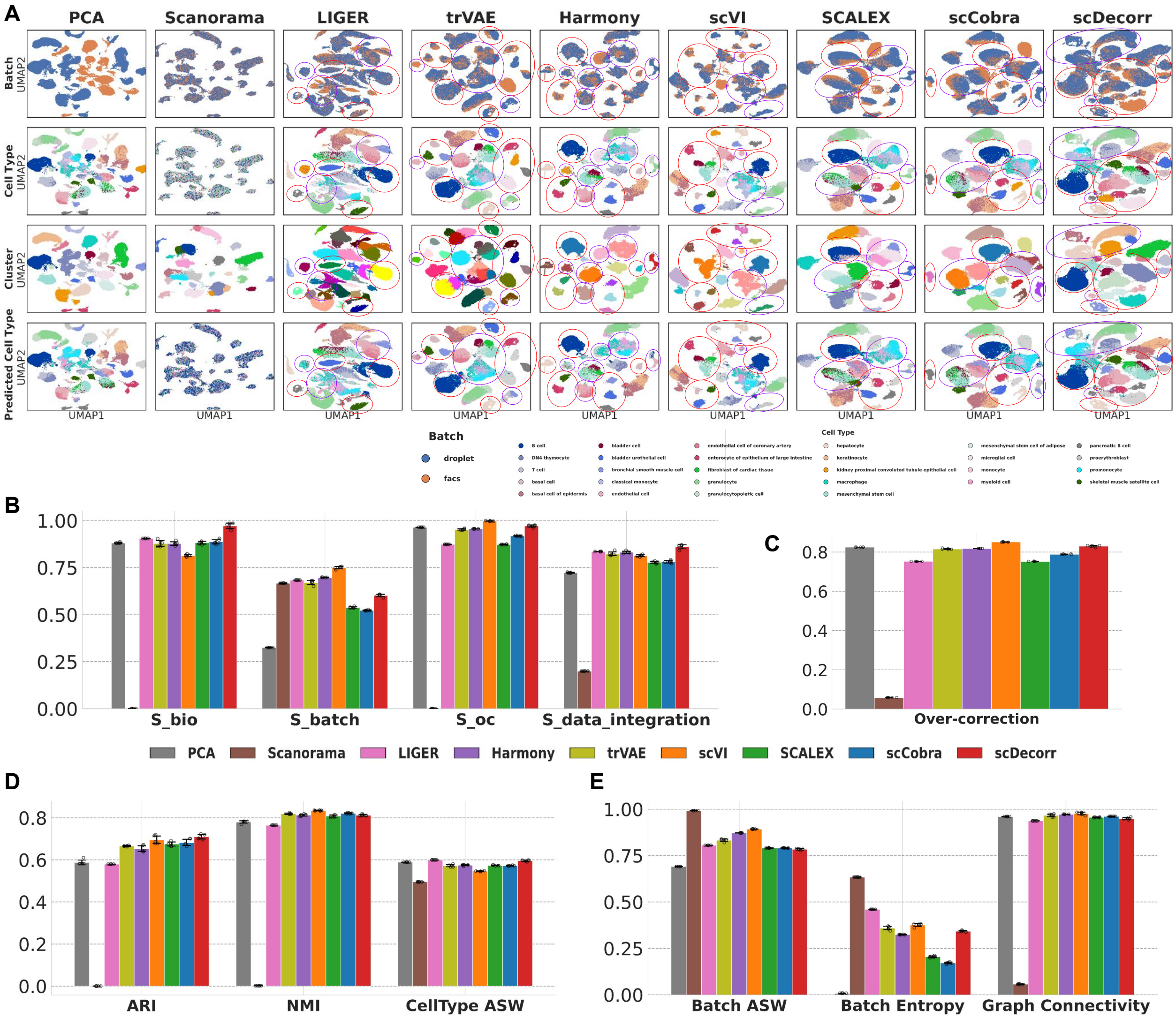

Figure S14: Data integration results on the tabula muris dataset. A. UMAP visualizations of benchmark method embeddings annotated with batch, author annotated cell-type, cluster and predicted cell-type labels (using inter-batch label transfer), B. Aggregated data integration scores of all benchmark methods visualized as barplots. Barplots visualizing raw over-correction (C), biological conservation (D) and batch correction (E) scores of all benchmark methods respectively.

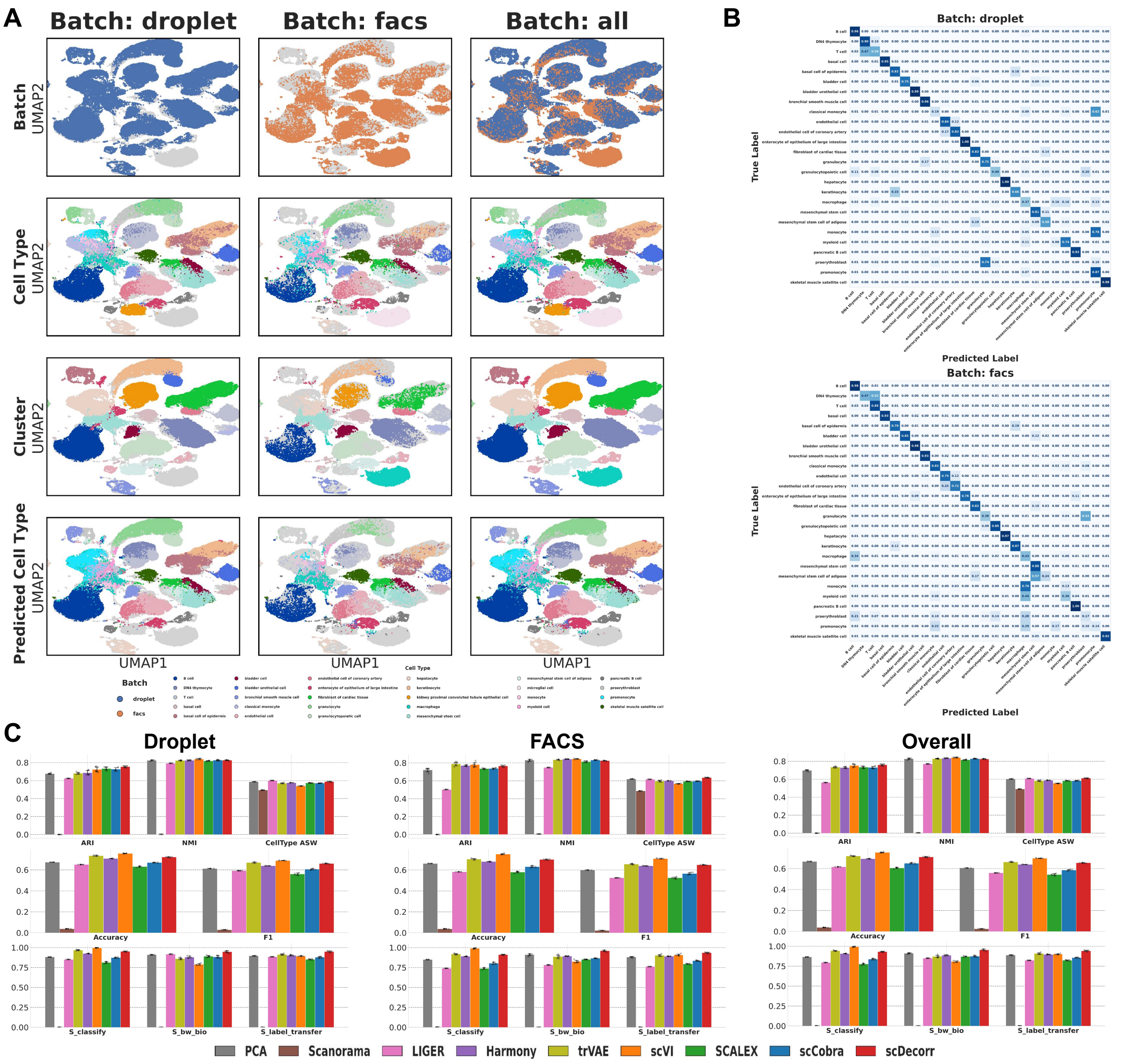

Figure S15: Label transfer results on the tabula muris dataset. A. UMAPs visualizing batch-wise predicted cell-types by scDecorr using other batches as reference. The plots also include batch-labels, author annotated cell-types, and cluster labels. B. Confusion matrices showing TPR (recall) of label transfer classification performance in every batch. C. Barplots showing batch-wise label transfer performance scores achieved by all benchmark methods. Error bars indicate variance of scores across 5 independent runs.

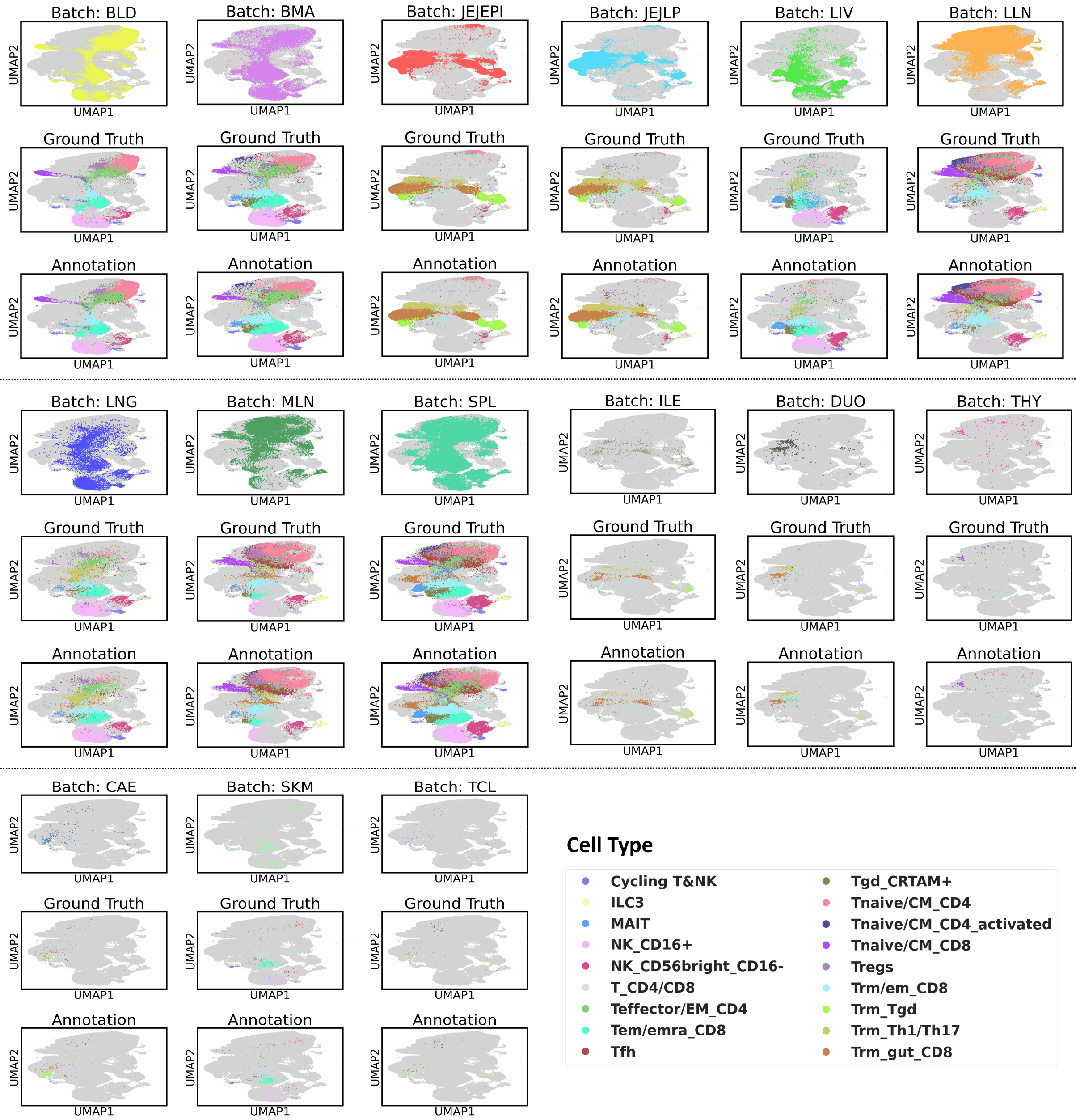

Figure S16: Cross-tissue Immune: UMAP plots of inter-organ label transfer using scDecorr for each organ in the dataset. Ground Truth refers to the annotations provided by the authors in their original study.

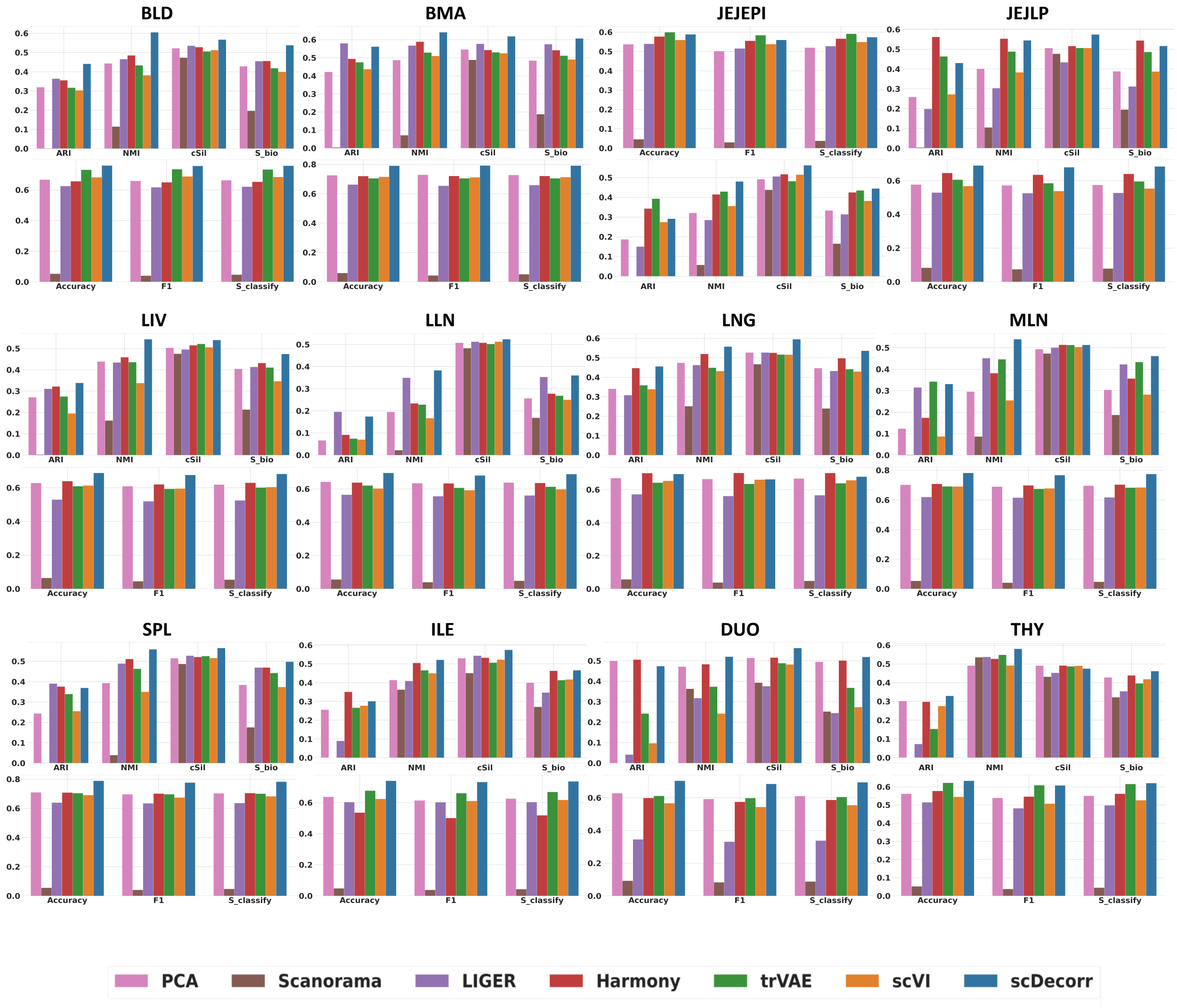

Figure S17: Cross-tissue Immune: Benchmark results of inter-organ label transfer. For each method, prediction accuracy measures (Accuracy, F1 and $S_{classify}$), clustering metrices such as NMI and ARI and bio conservation scores S_{bio} are provided for each organ.

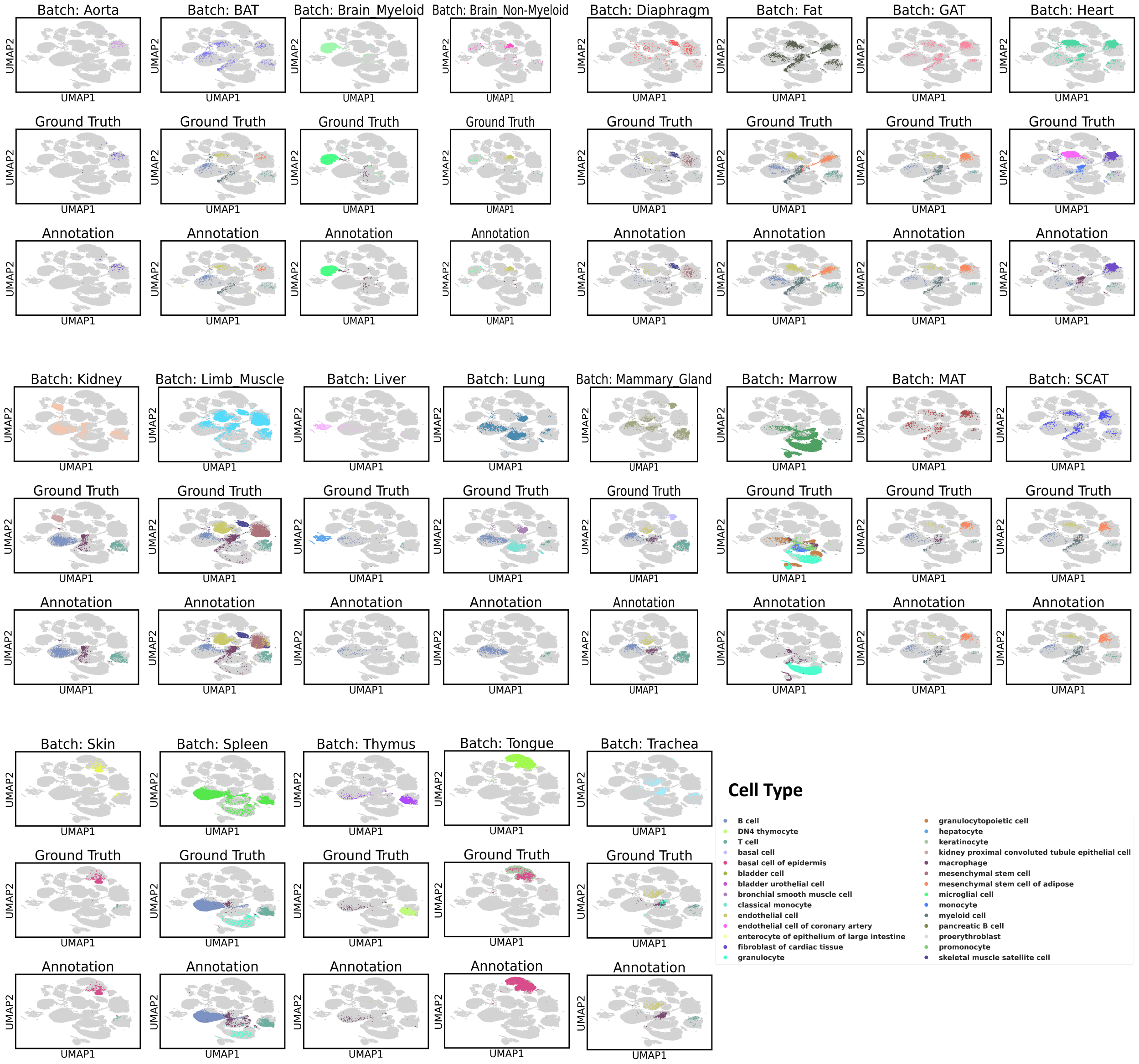

Figure S18: Tabula Muris: UMAP plots of inter-organ label transfer using scDecorr.

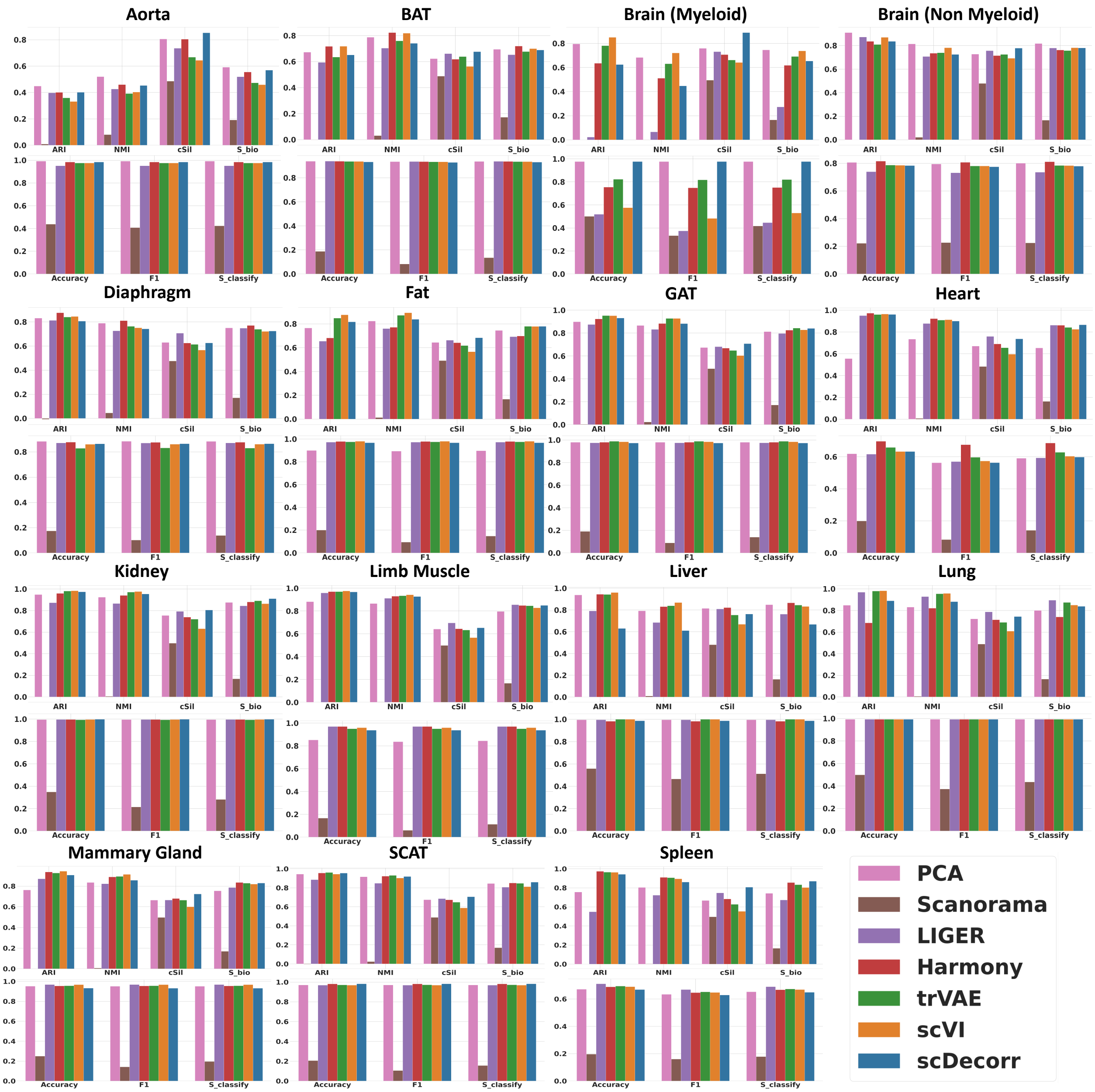

Figure S19: Tabula Muris: Benchmark results of inter-organ label transfer. Prediction and cluster accurcy metrices for different methods used in this study show the utility of embeddings generated by scDecorr for downstream tasks.

References

[1] C Dominguez Conde, C Xu, LB Jarvis, DB Rainbow, SB Wells, T Gomes, SK Howlett, O Suchanek, K Polanski, HW King, et al. Cross-tissue immune cell analysis reveals tissue-specific features in humans. *Science*, 376(6594):eabl5197, 2022.

[2] Malte D Luecken, Maren Buttner, Kridsadakorn Chaichoompu, Anna Danese, Marta Interlandi, Michaela F Muller, Daniel C Strobl, Luke Zappia, Martin Dugas, Maria Colome-Tatche, et al. Benchmarking atlas-level data integration in single-cell genomics. *Nature methods*, 19(1):41–50, 2022.

[3] Tabula Muris Consortium, Overall coordination Schaum Nicholas 1 Karkanias Jim 2 Neff Norma F. 2 May Andrew P. 2 Quake Stephen R. quake@ stanford. edu 2 3 f Wyss-Coray Tony twc@ stanford. edu 4 5 6 g Darmanis Spyros spyros. darmanis@ czbiohub. org 2 h, Logistical coordination Batson Joshua 2 Botvinnik Olga 2 Chen Michelle B. 3 Chen Steven 2 Green Foad 2 Jones Robert C. 3 Maynard Ashley 2 Penland Lolita 2 Pisco Angela Oliveira 2 Sit Rene V. 2 Stanley Geoffrey M. 3 Webber James T. 2 Zanini Fabio 3, and Computational data analysis Batson Joshua 2 Botvinnik Olga 2 Castro Paola 2 Croote Derek 3 Darmanis Spyros 2 DeRisi Joseph L. 2 27 Karkanias Jim 2 Pisco Angela Oliveira 2 Stanley Geoffrey M. 3 Webber James T. 2 Zanini Fabio 3. Single-cell transcriptomics of 20 mouse organs creates a tabula muris. *Nature*, 562(7727):367–372, 2018.

[4] Wenkai Han, Yuqi Cheng, Jiayang Chen, Huawen Zhong, Zhihang Hu, Siyuan Chen, Licheng Zong, Liang Hong, Ting-Fung Chan, Irwin King, Xin Gao, and Yu Li. Self-supervised contrastive learning for integrative single cell rna-seq data analysis. *Briefings in Bioinformatics*, 23(5):bbac377, 09 2022.
